# Diving into drug-screening: zebrafish embryos as an *in vivo* platform for antimicrobial drug discovery and assessment

**DOI:** 10.1093/femsre/fuae011

**Published:** 2024-04-29

**Authors:** Eva Habjan, Gina K Schouten, Alexander Speer, Peter van Ulsen, Wilbert Bitter

**Affiliations:** Department of Medical Microbiology and Infection Control, Amsterdam UMC, Location VU Medical Center,De Boelelaan 1108, 1081 HZ Amsterdam, The Netherlands; Department of Medical Microbiology and Infection Control, Amsterdam UMC, Location VU Medical Center,De Boelelaan 1108, 1081 HZ Amsterdam, The Netherlands; Department of Medical Microbiology and Infection Control, Amsterdam UMC, Location VU Medical Center,De Boelelaan 1108, 1081 HZ Amsterdam, The Netherlands; Section Molecular Microbiology of A-LIFE, Vrije Universiteit, De Boelelaan 1085, 1081 HV Amsterdam, The Netherlands; Department of Medical Microbiology and Infection Control, Amsterdam UMC, Location VU Medical Center,De Boelelaan 1108, 1081 HZ Amsterdam, The Netherlands; Section Molecular Microbiology of A-LIFE, Vrije Universiteit, De Boelelaan 1085, 1081 HV Amsterdam, The Netherlands

**Keywords:** antimicrobials, *Danio rerio*, zebrafish embryo, infection models, drug screening, animal models

## Abstract

The rise of multidrug-resistant bacteria underlines the need for innovative treatments, yet the introduction of new drugs has stagnated despite numerous antimicrobial discoveries. A major hurdle is a poor correlation between promising *in vitro* data and *in vivo* efficacy in animal models, which is essential for clinical development. Early *in vivo* testing is hindered by the expense and complexity of existing animal models. Therefore, there is a pressing need for cost-effective, rapid preclinical models with high translational value. To overcome these challenges, zebrafish embryos have emerged as an attractive model for infectious disease studies, offering advantages such as ethical alignment, rapid development, ease of maintenance, and genetic manipulability. The zebrafish embryo infection model, involving microinjection or immersion of pathogens and potential antibiotic hit compounds, provides a promising solution for early-stage drug screening. It offers a cost-effective and rapid means of assessing the efficacy, toxicity and mechanism of action of compounds in a whole-organism context. This review discusses the experimental design of this model, but also its benefits and challenges. Additionally, it highlights recently identified compounds in the zebrafish embryo infection model and discusses the relevance of the model in predicting the compound’s clinical potential.

## Teaser

This review examines the zebrafish embryo infection model for screening antimicrobial compounds, offering insights into its current status, benefits, challenges, and translational value in the context of antimicrobial drug discovery.

## Introduction

The introduction of antibiotics resulted in a decline in the global mortality rate of bacterial infections over the last century and has been aptly coined the most important medical discovery ever (Armstrong et al. 1999). However, the widespread use of antibiotics resulted in the inevitable increase in infections caused by multidrug-resistant (MDR) bacteria as previously reviewed (Organization 2015, Khan et al. 2017, Durao et al. 2018, Khawbung et al. 2021). Especially the rapid emergence of MDR isolates of *Acinetobacter baumannii, Pseudomonas aeruginosa, Enterobacteriaceae*, and *Mycobacterium tuberculosis* causes great concern (WHO 2017a, StopTBPartnership 2019). Despite efforts to stimulate the research for novel antimicrobial drugs, the discovery of drugs with a new mode of action has stagnated over the last 30 years (Silver 2011, Fair and Tor 2014, WHO 2017b, StopTBPartnership 2019). In fact, out of the hundreds of antibiotic hit compounds tested since 2017, only 11 have been admitted to the market. Moreover, nine out of these 11 belong to existing classes of antibiotics to which resistance mechanisms could already be present in clinical isolates (WHO 2020). One of the bottlenecks in introducing new compounds is that the *in vitro* data does not always translate to the compound’s efficacy in animal models or into favorable clinical outcomes (Aspatwar et al. 2017, Habjan et al. 2021, Schouten et al. 2022). Clearly, there is a need for preclinical models with a higher translational prediction value. Early compound testing using *in vivo* models would allow for the selection of *in vivo* active antimicrobials at an early stage of the drug discovery pipeline to increase the chances of also finding activity in clinical models. However, *in vivo* testing is often performed using mouse models, which are elaborate, expensive, and raise ethical issues, even more so when done in a medium or a high-throughput format. Therefore, a restricted number of antimicrobial compounds can be tested *in vivo* based on initial *in vitro* results, which consequently restricts the number of compounds that reach clinical studies (Koul et al. 2011, O’Neil 2016). Such an approach will fail to identify drugs that are not promising in the initial phase, but which show great activity *in vivo*, such as pyrazinamide. To circumvent these issues, zebrafish embryos have become an extensively used model to study infectious diseases (Van der Sar et al. 2004, Gomes and Mostowy 2020). In such a model, zebrafish embryos are infected with a pathogen of interest, usually through microinjection. Next, the infection, often lethal to the zebrafish, is followed over a period of a maximum of 5 days. Putative antimicrobial drugs can also be added to the water or can be injected once the infection is established. The efficacy of these compounds can be measured by assessing their effect on bacterial burden or zebrafish survival. In some cases, e.g. when using *Mycobacterium marinum*, the model allows for automated and robotized infection step, thus increasing experimental throughput by enabling testing many compounds (Spaink et al. 2013, Habjan et al. 2021).

Zebrafish embryos are attractive alternatives for other *in vivo* animal models. Firstly, during the first 120 h postfertilization (hpf), they are not considered experimental animals, and, therefore, alignment with an ethical committee is not required (see below for a detailed description). Furthermore, they show rapid embryonic development, high rates of proliferation, and small size and their maintenance is easy and low-cost, which contributes to their attraction as host models for infection studies. These characteristics, as well as the ease of genetic manipulation of zebrafish, have been extensively discussed in previous reviews (Sassen and Koster 2015, Neely 2017, Stream and Madigan 2022). In recent years, the value of the zebrafish embryo model has been widely recognized and reviewed in the literature in the fields of immunology (Ortiz et al. 2022, Stream and Madigan 2022), infectious diseases (Takaki et al. 2012, Takaki et al. 2013, Van Leeuwen et al. 2015, Varela et al. 2017, Gaudin and Goetz 2020, Linnerz and Hall 2020, Rasheed et al. 2021), oncology (Astell and Sieger 2020), toxicology (Brito et al. 2022), and developmental biology (Roper and Tanguay 2018), also including personalized medicine (Baxendale et al. 2017, Costa et al. 2020, Ochenkowska et al. 2022). Additionally, the zebrafish embryo infection model is emerging as an *in vivo* model to screen for novel antimicrobial drugs (Benard et al. 2012, Takaki et al. 2012, Habjan et al. 2021). The zebrafish embryo model, like other animal models, offers a platform to investigate compound activity and safety profiles. Their rapid development and optical transparency enable real-time visualization of drug responses, allowing early identification of safety concerns. Moreover, zebrafish embryos possess conserved metabolic pathways, allowing the assessment of certain pharmacokinetic (PK) and pharmacodynamic (PD) properties at the early stage of the research. This is significant since toxicity and insufficient PK/PD profiles are common factors contributing to early compound failures. Consequently, zebrafish embryos present a valuable tool for predicting antibacterial compound activity and mitigating early failures in drug research and development.

Here, we will describe the use of zebrafish embryos as an *in vivo* infection model to screen for compounds during the early stages of antimicrobial drug discovery. Furthermore, we will highlight what needs to be considered when using the zebrafish infection model and discuss its translational value, which we define here as the predictability of the outcome of the *in vivo* zebrafish embryo experiments for further clinical studies. To use the zebrafish model to its full potential, it is important to be aware of the benefits, challenges, and basic methods of this model. We will, therefore, first discuss such considerations.

## Regulatory considerations in zebrafish embryo research

In the European Union, animal research is regulated under Directive 2010/63/EU on the protection of animals used for scientific purposes (EC 2010). The directive provides regulations to implement an ethical approach to the use of animals in scientific research and is based on the 3Rs principle of Replacement, Reduction, and Refinement. According to the Directive, embryonic stages of zebrafish are considered a replacement or refinement since these developmental stages are likely to experience less or no pain, suffering, distress, or lasting harm when compared to adult animals (Strahle et al. 2011). This means that scientists are able to experiment on zebrafish as long as they are considered embryos. However, this experimental window for *in vivo* experiments depends on national regulations and definitions of the embryonic stages of zebrafish. In the USA, e.g. the definition of a zebrafish embryo is based on the time of hatching, a general rule for egg-laying species. Since zebrafish lay eggs, they are considered embryos until hatching, which typically occurs around 72 hpf, also indicated as 3 days postfertilization (dpf) (Bartlett and Silk 2016); after this timepoint, they are officially considered to be larvae. This definition can pose a challenge for scientific research since some protocols require dechorionation, which is the manual hatching of the embryos from their chorion before they hatch naturally. The question then arises if this is considered an artificial ending of the embryonic stage and, consequently, changes the legal time-span in which scientists can conduct their experiments. In the Animal Care and Use Committee (AMAC) guidelines of the NIH, there is a special category for zebrafish larvae that are younger than 7 dpf, because, in this period, the brain development has not yet reached a point where they can experience noxious stimuli. As such, experimentation with zebrafish larvae is more easily granted until 7 dpf. In Europe, zebrafish are considered embryos until they become capable of independent feeding, as reviewed in (Strahle et al. 2011). In zebrafish, this is accepted to be at 120 hpf (or 5 dpf), when both uptake and processing of external food start (Kimmel et al. 1995, Ng et al. 2005). Other countries usually follow either the American or the European rules, but it is advised to check this.

## Experimental design in zebrafish embryo infection models

The zebrafish embryo infection model is typically used to evaluate two fundamental characteristics of potential antimicrobial drugs: *in vivo* toxicity and *in vivo* activity. Various pathogenic bacteria can be used for infections, and different classes of antimicrobials can be evaluated. There are also several routes of administration of bacteria and compounds, and the choice depends on the aim of the study, the pathogen, i.e. used, and the physiochemical properties of the tested compounds. Previous investigations have explored and documented instances where variations in administration techniques have been found to influence pathogen virulence (Li and Hu 2012) or the activity of compounds (Fries et al. 2023) in the zebrafish embryo model.

## Administration routes of infecting bacterial pathogen in zebrafish embryo infection models

**Figure 1.**
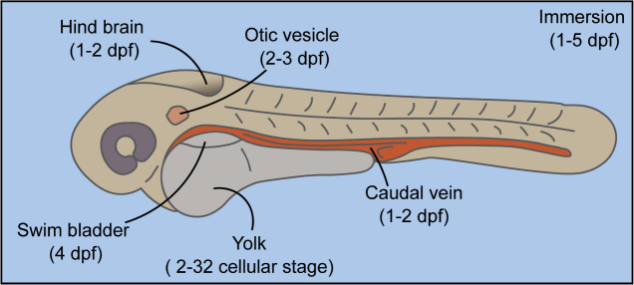
Administration routes for bacterial pathogens to establish a zebrafish embryo infection model. The illustration represents a zebrafish embryo at 48 hpf and shows locations and organs at which bacterial pathogens can be administered via microinjection. The time in days post fertilization (dpf) at which microinjection or immersion of pathogens can be performed is shown below the methods of administration.

There are two methods to introduce bacterial pathogens into zebrafish embryos: immersion, involving the addition of embryos to a solution containing bacteria, and microinjection (Fig. 1). For drug-screening purposes, a large number of infected embryos is required, making the immersion of zebrafish embryos a suitable route, as it is the least labor-intensive. During immersion, zebrafish embryos are immersed in a bacterial suspension (Sullivan et al. 2017) and successful infection results from the uptake of bacteria either via percutaneous absorption or via oral uptake (Van Soest et al. 2011). The latter is only possible at ~3 dpf when the mouth of the embryo opens, which significantly reduces the experimental window, as previously reviewed (Singleman and Holtzman 2014). Moreover, high doses of bacteria, ranging from 10^8^ to 10^9^ bacteria per ml, are needed to establish an infection via immersion (Van Soest et al. 2011, Varas et al. 2018). Another disadvantage of the method is that it is impossible to control the number of pathogens, i.e. taken up by each embryo, thereby increasing variation and reducing the statistical significance. To facilitate the uptake of the pathogen using the immersion method, it is possible to use tail-injured embryos, where a needle is used to achieve a small transection in the tail of embryos (Nogaret et al. 2021). Because of the variability of the immersion method, it has only been used to infect zebrafish embryos with a few pathogens, such as *Edwardsiella tarda* (Pressley et al. 2005, Van Soest et al. 2011), *Flavobacterium columnare* (Chang and Nie 2008), *Lactobacillus paracasei* (Toh et al. 2013), *Eubacterium limosum* (Toh et al. 2013), *Listeria monocytogenes* (Shan et al. 2015), *P. aeruginosa* (Pont and Blanc-Potard 2021), and *Salmonella enterica spp typhimurium* (Varas et al. 2018). An advantage of immersion as an infection method is that this uptake route may be closer to natural infection routes.

Microinjection is performed by using a glass microcapillary injection needle to inject a bacterial suspension into the embryo within 1 or 2 dpf (Van der Sar et al. 2003, Sullivan et al. 2017). The location of the injection depends on the pathogen of interest and disease, i.e. mimicked (see below) and can be intravenous or in tissues like the yolk, the hindbrain, the swim bladder, or otic vesicle as reviewed by Benard et al. (2012) and Sullivan et al. (2017). Yolk microinjections are performed within a few hours after fertilization, whereas intravenous microinjections are possible from 1 dpf onwards. Injections to other injection sites, such as the hindbrain or the otic vesicle, require more time to develop and can be injected at 2 dpf. Since this method requires a precise injection into a specific organ of the zebrafish embryo, the method is labor-intensive and time-consuming, and thus unsuitable for high-throughput compound screening where large quantities of infected embryos are required.

Nevertheless, intravenous microinjection in the caudal vein is the most commonly used technique to infect zebrafish embryos with microorganisms since it allows precise and reproducible infectious dose, spread of infection throughout the whole organism and is suitable for a wide range of pathogens. Differences in dissemination, replication and clearance of individual bacteria can be tracked if a bacterial strain expressing a fluorescent protein is used. For example, by using fluorescent bacteria it has been shown that both *M. marinum* and *S. enterica spp typhimurium* are rapidly taken up from the bloodstream by embryonic macrophages. Importantly, the macrophages containing *M. marinum* leave the bloodstream and give rise to early granulomas in tissue, which is a pathological hallmark of tuberculosis (TB; Prouty et al. 2003, Lesley and Ramakrishnan 2008), while the macrophages loaded with *S. enterica* stay in the bloodstream until they are killed by the pathogen (Van der Sar et al. 2004).

As mentioned, it is possible to inject the bacteria at a compartmentalized site of the zebrafish embryo; as previously reviewed for the swim-bladder (Sullivan et al. 2017), the hindbrain (Jim et al. 2016, Stirling et al. 2020, Pont and Blanc-Potard 2021), or the otic vesicle (Harvie and Huttenlocher 2015, Pont and Blanc-Potard 2021). The advantage of these sites is that they are without direct access to the vascular system by the pathogen (Harvie and Huttenlocher 2015). Additionally, they both initially have a low number of immune cells, which gives the infection time to develop. Because of this, infection of either tissue is often used to study immune cell migration to the site of infection and the development of inflammatory cues, as reviewed by (Sullivan et al. 2017, Stirling et al. 2020, Pont and Blanc-Potard 2021). Injecting in such closed compartments allows better imaging of the infection (Harvie and Huttenlocher 2015). This method is especially useful for studying pathogens that affect the brain, such as *Streptococcus pneumoniae* (Jim et al. 2016, Pendergast et al. 2023). Of course, one should consider the relevance of this method, as the pathogens do not need to cross the blood–brain barrier. Additionally, similar to intravenous microinjection, the labor-intensive and time-consuming nature of the technique makes it unsuitable for high-throughput drug screening.

When higher numbers of infected embryos are needed, e.g. for drug screens, microinjection into the yolk can be an alternative for immersion. Here, the bacterial suspension is injected into the yolk of larvae at the 2–32 cellular stage (Benard et al. 2012). This method can only be done during early developmental stages to prevent harming the embryos (Li and Hu 2012, Fehr et al. 2015, Zaccaria et al. 2015). A great advantage for drug screening purposes is that a robotic system for automated yolk injection can be applied (Wang et al. 2007, Carvalho et al. 2011, Veneman et al. 2013, 2014, Habjan et al. 2021). This method works best with slow-growing bacteria, such as *M. marinum*, which spread from the yolk into the developing embryo and cause a systemic infection, resulting in the formation of early granulomas. In successful early stage drug discovery screens for anti-TB drugs, yolk infections were combined with the immersion of infected embryos with compounds to allow for hundreds of embryos per experiment (Ordas et al. 2015, Habjan et al. 2021). Unfortunately, thus far, it has not been possible to adapt this strategy to fast-growing pathogens, as these fast-growing species thrive in the nutrient-rich yolk and are usually lethal to the larvae within 12 h postinfection (hpi), even when treatment with established antibiotics is directly applied, and robotic caudal vein injection is still under development.

Another important consideration is that zebrafish embryos are maintained at temperatures ranging from 28°C to 30°C, which is not optimal for the growth and/or virulence of human pathogens like *M. tuberculosis, Escherichia coli, A. baumannii*, and *P. aeruginosa*, which typically thrive at 37°C. Incubating pathogens at lower temperatures could impact the outcomes of infection studies. To address this issue, researchers often use fish-related pathogens. As previously mentioned, a fish pathogen *M. marinum* serves as a suitable model for *M. tuberculosis*, offering a relevant alternative for infection studies. While zebrafish embryo infection with *M. marinum* mirrors many aspects of TB in humans, significant differences exist, as reviewed by Meijer et al. (2016). One of these differences is the route of infection. In humans, *M. tuberculosis* infects the lungs through inhalation of the pathogen, whereas in nature, the zebrafish probably gets infected via the gut, whereas in experimental design, the pathogen is injected via the bloodstream or yolk.

One of the major difficulties scientists encounter when working with the zebrafish embryo infection model is the variability between test animals and between experiments. One reason for this is that zebrafish are not clonal; therefore, even established zebrafish lines show variation between embryo batches. Another factor that plays a role is the rapid embryonic development of zebrafish, especially their immune system. Within 24 hpf, the first macrophages appear and after 48 hpf hours neutrophils and components of the complement system are present (Kimmel et al. 1995, Herbomel et al. 1999). Due to these rapid changes, the time of infection is decisive in the spread and replication of bacteria in the embryos (Veneman et al. 2013). For instance, the number of phagocytes is critical for the outcome of *P. aeruginosa* infection; as soon as there are enough phagocytes, this infection is kept under control, but depletion, or a too early injection, leads to a rapidly fatal infection (Brannon et al. 2009). Therefore, this requires expanded group sizes and multiple repetitions to obtain statistically significant differences between experimental groups. Different studies have employed varying numbers of embryos per experimental group. For instance, studies using a single bacterial strain with different compound treatments have utilized 5–10 embryos per treatment group (Ho et al. 2021, Winters et al. 2022), 12–15 embryos (Habjan et al. 2021, Schouten et al. 2022), as well as 20–30 (Ordas et al. 2015, Dalton et al. 2017, Goh et al. 2023) or even more than 30 zebrafish embryos per experimental group (Wambaugh et al. 2017). The optimal number may vary depending on experimental requirements and constraints. Ensuring adequate statistical power is vital for detecting meaningful differences or effects between experimental groups. Conducting a small pilot experiment for power analysis can provide insights into expected effect sizes, variability, and desired significance levels, aiding in determining the appropriate sample size. However, it is generally advisable to aim for a sample size of 15–20 embryos per experimental group with a minimum of three experimental repeats to detect meaningful statistical differences.

In addition, it is important to realize that the infection dose also significantly influences the severity and reproducibility of the infection within the zebrafish embryos. Suboptimal doses may lead to false negative results or inconsistent and unreliable outcomes, while excessively high doses might result in overwhelming infections that obscure mild effects. Therefore, it is crucial to perform calibrations of the infection dose using a titration study (Lee et al. 2015, Fries et al. 2023). Additionally, an established antibiotic in varying concentrations can be used as a control to identify the infection dose that shows a measurable response without overwhelming the zebrafish embryo immune system. Naturally, the optimal infection dose can vary depending on the route of infection. For example, Van Soest et al. (2011) showed that infection with *E. tarda* via static immersion results in mortality rates between 25%–75%, whereas intravenous injection leads to 100% mortality, demonstrating not only that static immersion leads to more variety in infection rate, but also that intravenous injection requires a smaller infection dose than static immersion to reach a reliable and measurable response.

## Administration routes of antibacterial compounds in zebrafish embryo models

The two main administration routes for compounds are again microinjection and immersion. Many compounds are dissolved in DMSO, which does not cause problems, as the fish can tolerate up to 1% DMSO in the medium (Hoyberghs et al. 2021) and up to 50% in the injection solution (Schouten et al. 2022). Usually, precipitation of the compound after dilution in those volumes poses a bigger problem.

Microinjection of compounds is a laborious and time-consuming technique. Commonly, compounds are injected when the infection in the zebrafish has had time to establish. For fast-growing bacteria, such as *E. coli* or *A. baumannii*, the time between infection and compound treatment is 1 h, whereas slow-growing bacteria, such as *M. marinum*, need more time and are usually treated with compounds 1 day past infection (Habjan et al. 2021, Schouten et al. 2022). The pathogen and the compound can also be coinjected, although this is considered less desirable as the pathogen may already be damaged or eradicated in the injection solution. An advantage of injection of the test compounds over immersion of the zebrafish embryos in compound-containing solutions is that it allows for precise control of the dose, i.e. added to the embryo.

The second drug administration route is the immersion of infected embryos in a solution containing a test compound. An advantage of immersion is that the embryos can be kept in the compound-containing solution for the total duration of the experiment, or the solution can be refreshed daily. Here, the compound is taken up passively through the skin and, after 72 hpf, via gills and the gut (Van Soest et al. 2011). Although it has been demonstrated that zebrafish can absorb molecules through their skin, the absorption varies considerably and, therefore, it remains a challenge to determine how much of the compound is taken up (Van Soest et al. 2011, Benard et al. 2012, Dalton et al. 2017, Morikane et al. 2020). When treatment is performed before 48–72 hpf, the embryos are still in the chorion, which can further impact the compound’s uptake (Henn and Braunbeck 2011, De Koning et al. 2015, Chen et al. 2020). To overcome this issue, either enzymatic dechorionation of embryos with Pronase (Henn and Braunbeck 2011) or manual dechorionation can be performed. The immersion method is an easy and quick treatment route and, therefore, especially attractive when testing many compounds (Ordas et al. 2015, Habjan et al. 2021).

**Figure 2.**
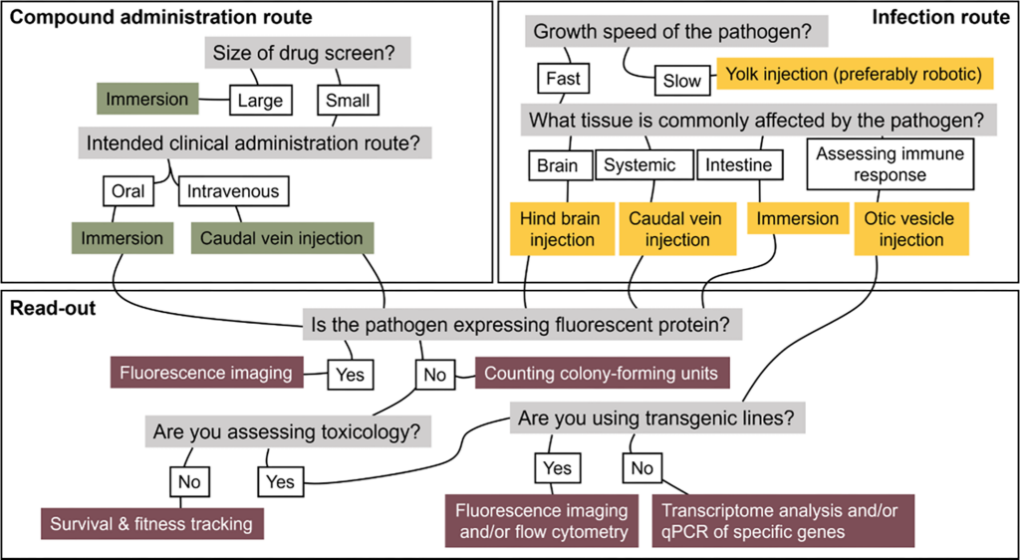
Decision tree for determining the appropriate method for administration of test compounds, infection route, and read-out when using the zebrafish infection model for antimicrobial compound screening.

In addition, it is crucial to consider how the administration of compounds affects the model’s predictability. Compounds with poor oral bioavailability are preferably injected into the zebrafish bloodstream rather than administered via immersion. This ensures a more accurate and clinically relevant representation of drug absorption and distribution, enhancing the relevance of the zebrafish embryo model. Thus, it is imperative to choose the correct method to administer both the test antimicrobials and the pathogen. To facilitate an informed choice on these three aspects, the decision tree in Fig. 2 can be consulted.

## Follow-up methods for tracking bacterial infection and assessing antibacterial activity in the zebrafish embryo model

**Figure 3.**
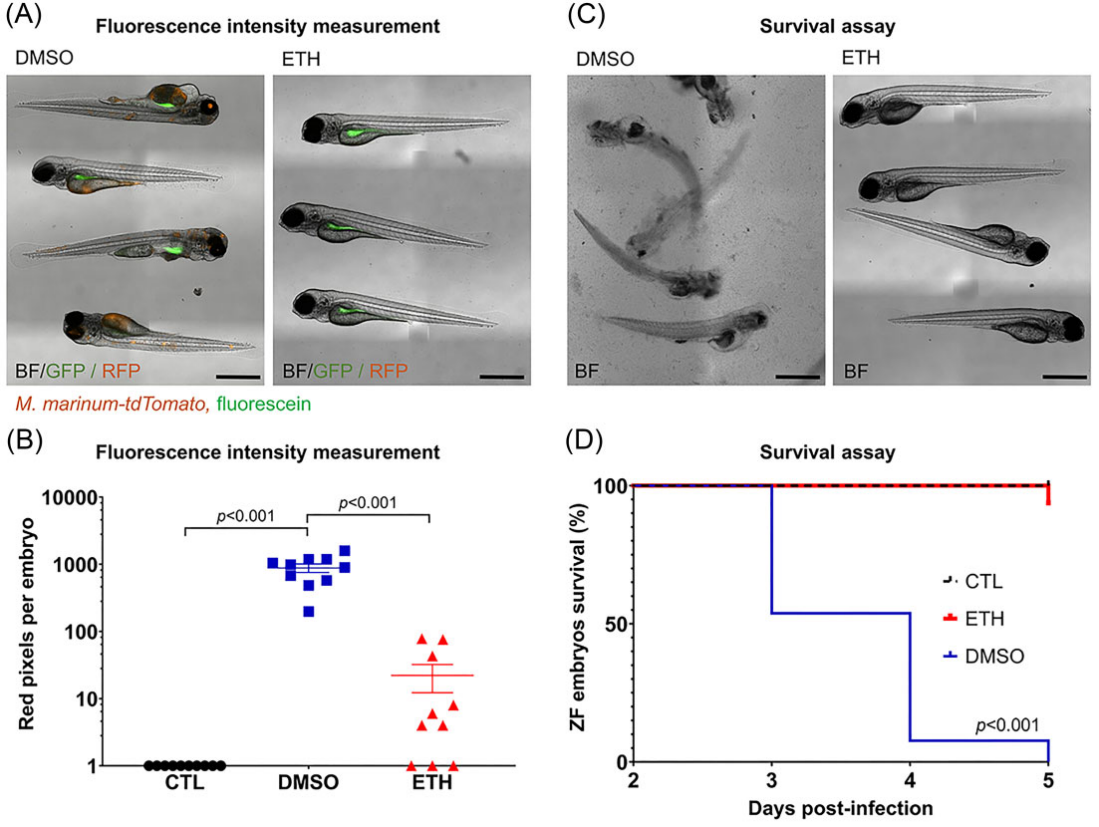
Comparison of zebrafish infection model follow-up methodologies. (A) and (B) Zebrafish embryos were infected via yolk microinjection with 100 CFU of *M. marinum* M expressing the red-fluorescent protein tdTomato, mixed with the green fluorescent dye. Treatment with DMSO or Ethionamide (ETH, 1 µM) was administered 1 dpf via immersion. Each treatment group consisted of 10 embryos. On the 5th dpf, the embryos were imaged using fluorescence microscopy (A), the scale bar on the images represents 500 µm. The integrated red fluorescent signal per embryo was used to quantify the bacterial load (B). Each dot on the graph represents the total red fluorescence signal per embryo. Statistical significance was determined by one-way ANOVA, following Dunnett’s multiple comparison test by comparing the signal from the DMSO-treated control sample with each treatment group (^****^P ≤ .0001). (C) and (D) Embryos were yolk-infected with 1000 CFU of *M. marinum* M and treated via immersion with DMSO or ETH (1 µM) 1 dpi. Each treatment group consisted of 10 embryos. Images of embryos were taken on the 5th dpf (C), the scale bar represents 500 µm. The daily zebrafish (ZF) embryo survival was determined based on the presence of a heartbeat. The Kaplan–Meier survival tests were conducted to generate the survival curves (D), and *P*-values were calculated by the log-rank test. The “CTL” group represents the noninfected control group.

The zebrafish embryo infection model allows for several readouts when screening for antibiotic hit compounds (Table 1, Fig. 3). The most commonly used readout is tracking the survival of infected embryos over time. Survival can be easily followed by determining the presence of a heartbeat. This is an unambiguous readout that can even be automated (Kang et al. 2018, Gierten et al. 2020, Santoso et al. 2020). However, two major disadvantages of this method are that it does not provide information on the localization of the bacteria in the embryo and that there can be high variability between the batches of embryos. Furthermore, the infection must be lethal to allow for survival to be used as a measuring factor. For example, only about one in five of the clinical *E. coli* and *A. baumannii* strains raise a lethal infection (Schouten et al., in press). Furthermore, slow-growing pathogens usually are not able to overwhelm the zebrafish within the short time frame of the experiments but do increase in numbers within the fish. Two alternatives to follow the ongoing infection and the effectivity and toxicity of compounds are tracking the general fitness of embryos and determining the bacterial load, which both can be adapted to high-throughput formats.

**Table 1.** An overview of follow-up methodologies, along with their respective advantages and disadvantages, employed in zebrafish embryo infection models to evaluate antimicrobial efficacy.

Follow-up method	Advantages	Disadvantages
Zebrafish Survival tracking	• Straightforward, does not require expertise or special equipment • Unambiguous readout • Can be automated	• Limited to lethal infections • Does not provide information on bacterial localization
Zebrafish fitness tracking	• Provides insight into overall embryo health and development • Correlates with infection severity	• Interpretation may be complicated by compound toxicity
Bacterial load assessment by CFU counting	• Quantitative measurement of infection	• Labor-intensive process • Risk of contamination from commensal bacteria • Sacrifices embryos for assessment, limiting longitudinal studies
Bacterial load assessment by fluorescence measurement	• Quantitative measurement of infection • Real-time visualization of infection progress • Compatible with high-throughput screening • Can be automated	• Limited to fluorescence-based assays • Signal may persist after bacterial death • Signal dispersion and tissue quenching may lead to underestimation
Bacterial load assessment by COPAS	• Quantitative measurement of infection • Can visualize and quantify infection in different regions of the embryo (e.g. head, body, tail, and yolk)	• Requires specialized equipment
Bacterial load assessment by fluorimetry	• Automated • Quantitative measurement of infection • Suitable for analyzing of large quantity of infected embryos	• Signal may persist after bacterial death • Signal dispersion and tissue quenching may lead to underestimation • Limited to fluorescence-based assays
Automated image analysis	• Reduces labor intensity and human error • Software adaptable to experimental needs	• Requires diverse dataset for effective machine learning

The general fitness of embryos can be tracked using developmental markers, e.g. nondetachment of the tail, lack of somite development, lack of swim bladder development, the appearance of necrotic tissue, and heart edema (European Commision 2013). Impaired development of these features in zebrafish embryos seems to be proportional to the severity of an infection. However, it should be noted that such developmental changes can also be caused by the toxicity of the test compound, complicating the interpretation of results when testing the activity of antimicrobial compounds.

Probably the most accurate method of analyzing an ongoing infection and the efficacy of an added compound is to determine bacterial load. The bacterial load can be either assessed by counting colony-forming units (CFU) or by measuring fluorescence signals from bacteria expressing a fluorescent protein (Basheer et al. 2020). To determine CFU count, infected zebrafish embryos are lysed after treatment and plated on selective agar. This approach is labor-intensive and the presence of commensal bacteria in the gut and on the surface of the zebrafish is a high liability to contaminate the plates. Moreover, since the embryos are sacrificed, assessing treatment efficacy over time requires considerable amounts of zebrafish embryos, rendering this method also less compatible with high-throughput drug screens. Therefore, assessing fluorescence as a measurement of infection is more practical. By using bacterial strains that express fluorescent proteins, embryos can be imaged using a fluorescence microscope at different time points after infection to visualize and quantify the ongoing infection and differences in activity between compounds (Takaki et al. 2012). An interesting alternative approach is using bioluminescently tagged pathogen, which allows rapid, real-time measurement of signal only from viable pathogens (Dalton et al. 2017). In order to follow the bacteria for several days, zebrafish embryos that stay transparent for a longer duration are most suited. This transparency was accomplished by genetically mutating the embryos, resulting in a lack of pigment (White et al. 2008). This transparent zebrafish line, called the *Casper* line, was established by combining the spontaneous mutations in the *roy orbison* zebrafish line, in which the zebrafish lack iridophores, lack pigmented eyes and have translucent skin, with a mutation in the *mifta* gene, which renders the zebrafish completely absent of melanocytes (White et al. 2008). Alternatively, zebrafish embryos can be continuously chemically treated with 1-phenyl 2-thiourea (PTU) to inhibit pigment formation (Karlsson et al. 2001). Note, however, that the measurement of fluorescence at different time points is rarely done using the same zebrafish embryos since the anesthesia required for the imaging procedure can negatively influence the fitness of the zebrafish embryo and, thus, the outcome. Readouts can be further modified by using a transgenic fish line that expresses a fluorescent marker in a specific tissue or cell type to look at the interaction of bacteria with specific host cells (Takaki et al. 2012, Jim et al. 2016, Van Leeuwen et al. 2018, Miskolci et al. 2022). Some concerns have been raised about how well fluorescence correlates to the severity of infection. Because fluorescent proteins are generally very stable (Stepanenko et al. 2004, Scott et al. 2018), their signal may still be detected even when the bacteria are dead. Moreover, the fluorescent signal can be dispersed and, thus, underestimated, especially if imaging is done only in a single Z-plain and not as Z-stack. The embryo is a three-dimensional organism and measuring the signal only at a specific depth might not represent the overall signal. Another challenge is a differential quenching of fluorescence by the different tissues. Because of these concerns, fluorescence measurement can be combined with determining both the CFU count and the survival of the embryos and studies have shown that these three measurements strongly correlate (Stoop et al. 2011, Stirling et al. 2020, Knudsen Dal et al. 2022). As a result, fluorescence is nowadays established as a major readout of infection load (Table 1).

The fluorescence readout is especially suitable for high-throughput applications since image acquisition and analysis can be automated. In our previous experiments, we used the software package Cell Profiler (Lamprecht et al. 2007), with which each zebrafish embryo in the picture can be manually selected to measure the integrated pixel intensity of the fluorescent signal per embryo (Phan et al. 2018, Van Leeuwen et al. 2018). However, manual selection, involving encircling each embryo, is labor-intensive and prone to mistakes. Thus, we have adapted the program to include an in-house module, developed using machine learning, that selects and encircles zebrafish embryos automatically (Habjan et al. 2021). The machine learning was done with a diverse data set, including dead and deformed zebrafish embryos. Therefore, the module can categorize the selected embryos into different categories, e.g. dead, alive, and deformed. Consequently, we are able to score a compound’s activity as well as its lethality/toxicity, which increases the usefulness of the method for selecting interesting hits.

To allow for discrimination between injected and noninjected embryos, which is especially useful if automated injection is used, the green fluorescent dye fluorescein can be added to the pathogen mixture. This enables the software to select only successfully injected embryos based on the presence of green fluorescence (Habjan et al. 2021). Several other methods have been reported to streamline and simplify the read-out procedure and increase the throughput of the analysis. Takaki et al. (2012) developed an automated 96-well plate fluorimetry assay using a plate reader, in which fluorescence corresponds to the relative bacterial number in infected zebrafish embryos per well. Another approach is using a Complex Object Parametric Analyzer and Sorter (COPAS) flow cytometry (Carvalho et al. 2011, Veneman et al. 2014). It is based on a continuous flow system that can analyze large quantities of objects using five parameters: size, optical density, and up to three channels of fluorescence. Quantification can be expressed as an average fluorescence per embryo or individual profiles for each analyzed embryo can be generated, where the distribution of the labeled object can be seen in different regions, e.g. head, body, tail, and yolk.

## Absorption, distribution, metabolism, excretion (ADME), and toxicity assessment of antibacterial compounds in zebrafish embryos

The zebrafish model has recently been further developed to test important characteristics, like the ADME properties of compounds. The effective internal drug concentration is a crucial parameter in evaluating compound activity in animal models, since compounds with poor ADME properties might not reach target sites in sufficient concentrations to exert their desired effects. This could consequently yield false negative results despite a compounds potential antimicrobial activity. Therefore, it is important to consider what the effect of the infection route is on the compound. For example, when immersion is used to treat infected zebrafish embryos, it is important to determine the compounds stability in water and evaluate the optimal exposure time of the infected zebrafish to the compound. Some compounds require longer exposure times to effectively combat the infection, leading to false negative results if the exposure period is too short. Moreover, instable compounds might need to be administered repeatedly to exert their antimicrobial effects. In addition, zebrafish embryos are kept at a temperature ranging between 28°C and 30°C, whereas most human pathogens have optimal growth at a temperature of 37°C. This difference in temperature can affect both the compounds activity and the virulence of the pathogen and thus lead to false positive results or false negative results. Furthermore, the age of the zebrafish embryo might also play a role in the optimal uptake of compounds. As described by Fries et al. (2023), when zebrafish embryos are immersed in compound solution to treat infections, they show increasing drug sensitivity with age, since at 30 hpf, the uptake of compounds depends solely on passive diffusion through the skin and is only complemented by uptake through the gastrointestinal tract after 72 hpf.

However, it is challenging to determine the internal concentration of a compound due to the embryos’ small size and low blood volume. Nevertheless, there have been reports of using nanoscale blood sampling (Van Wijk et al. 2019) in combination with liquid chromatography-tandem mass spectrometry (LC-MS/MS) to measure the internal drug concentrations (Zhang et al. 2015). The latter study confirmed that the uptake of compounds is highly dependent on the compound’s physiochemical properties. A comparison of zebrafish intrabody exposure of different fluorescent dyes after treatment via yolk-injections or immersion showed that the levels of lipophilic compounds inside the embryos were similar when treated via immersion or yolk-injection. Conversely, intrabody levels of more hydrophilic compounds were extremely low after immersion; thus, microinjection of such compounds is recommended (Guarin et al. 2021). Conversely, another study has shown that the compound’s hydrophobicity negatively influences uptake levels when the compound is administered via immersion (Ordas et al. 2015). The fact that hydrophilic compounds are more likely to be orally active drugs also aligns with Lipinski’s Rule of Five (RO5), which delineates molecular characteristics crucial for oral drug PKs in humans. The general guidelines of RO5 include a molecular mass (Mw) less than 500 Da, no more than five hydrogen bond donors, no more than 10 hydrogen bond acceptors, and an octanol–water partition coefficient log P (clogP) not exceeding 5. To answer the question if zebrafish-active compounds obey Lipinsk’s RO5, Long et al. (2019) investigated the parameters of 700 chemicals previously active in zebrafish infection models via immersion, revealing that zebrafish-permeable compounds typically fall within the molecular weight range of 200–500 Da, but they tend to be more lipophilic, with a clogP ≤ 5.3. Several other studies align with Lipinski’s rule; for instance, Linezolid showed activity via immersion in the zebrafish infection model, and Linezolid is a lipophilic compound (clogP = 0.55) with low molecular weight (Mw: 337.35 g/mol) (Fries et al. 2023). Conversely, small hydrophilic antibiotics like ciprofloxacin, tetracycline, and cefazolin only displayed activity when injected into the embryo, not via immersion (Fries et al. 2023). This observed inactivity of compounds with molecular weight and lipophilicity that fit Lipinnski’s RO5 may relate to the compounds’ polarity, as it was previously reported that ionic compounds display hindered diffusion (Brox et al. 2016).

Two recent studies measured the uptake of isoniazid or paracetamol after bathing the embryos in solutions containing these compounds. It was shown that the blood concentration of isoniazid was only 20% of the external drug concentration surrounding the fish, while this was even 10% for paracetamol (Van Wijk et al. 2019). Analyzing solely the blood levels of compounds in zebrafish embryos may impose limitations and potentially result in false negatives, given the rapid distribution or accumulation of compounds into tissues beyond the bloodstream, including fat tissue. The small size of embryos poses a considerable challenge for conducting tissue-specific studies, however, a sampling from yolk has been previously reported (Ordas et al. 2015). Moreover, emerging technological advancements, such as the matrix-assisted laser desorption/ionization mass spectrometry imaging (MALDI MSI) method in zebrafish embryos, can provide information into the distribution and metabolism of compounds (Asslan et al. 2021, Park et al. 2023). It is worth noting that these experiments are currently considered proof-of-principle studies and are not yet standard practice due to the labor-intensive nature of the procedures and the need for specialized equipment.

Importantly, tissue distribution in zebrafish seems to correlate with distribution in mammals (Liu and Wen 2002, MacRae and Peterson 2015, Cavalieri 2020, Blumenthal et al. 2021, Wang et al. 2021). To investigate tissue distribution in zebrafish, the compound could be linked with a fluorophore to track its location in the embryo, as has been done to track silica nanoparticles in zebrafish embryos (Sharif et al. 2012). However, such an addition could affect the antibacterial activity, ADME properties, and uptake of the compound by the zebrafish embryos, complicating the interpretation of experiments considerably.

How a compound is metabolized in the experimental animal also plays a significant role in the compound’s effectivity and the translational value of different models. For example, mice have a more rapid metabolism than humans, resulting in shortening the half-life of compounds and reducing their effect (Terpstra 2001). Interestingly, a recent study reported about a natural compound sorangicin A, which showed activity in *S. aureus-*infected rats and zebrafish embryos but was not effective in a mouse model due to fast degradation in plasma (Fries et al. 2023). Unfortunately, data comparing the metabolic rate between humans and zebrafish embryos is not available. Furthermore, not only the rate of metabolic turn-over, but also the level of conservation of the metabolic processes in the host is of great importance. In zebrafish embryos, both phase I (oxidation, *n*-demethylation, *o*-demethylation, and *n*-dealkylation) and phase II (sulfation and glucuronidation) metabolic processes found in mammals are present (Alderton et al. 2010, Diekman and Hill 2013). Although the enzymes belonging to both metabolic phases are highly conserved to the ones from mammals (Alderton et al. 2010, Diekman and Hill 2013), there have been reports of differences in their responses to compounds (Diekman and Hill 2013). Moreover, it should be kept in mind that the liver of zebrafish does not develop until 60–72 hpf and is only complete at 5 dpf (Chu and Sadler 2009). Thus, the way drugs are metabolized might significantly vary through the different developmental stages, and, therefore, the timepoint of adding a drug can influence the observed results (Saad et al. 2017, Verbueken et al. 2018). In addition, it was reported that the drug administration route can also influence the metabolic conversion of compounds (Park et al. 2020).

In mammals, the oxidases of the cytochrome P450 (CYP) family are mostly responsible for phase I metabolism. Zebrafish do possess CYP orthologues; however, the full extent of how conserved the metabolic processes are remains to be investigated (Patrzykat et al. 2001, Li and Hu 2012, MacRae and Peterson 2015). We do know that the zebrafish orthologue of CYP3A (CYP3A65), which plays a role in metabolizing 50% of all human drugs (Fukami et al. 2022), was shown to be expressed in the liver and intestine of larvae and adult zebrafish (Bresolin et al. 2005). Furthermore, adding rifampicin resulted in the upregulation of CYP3A65 expression. This resembles the situation in humans, where the addition of rifampicin upregulates CYP3A, but interestingly not in rats (Rubinstein 2006). Several other studies also showed functional parallels of CYPs and also non-CYP metabolic enzymes between humans and zebrafish (Liu and Wen 2002, MacRae and Peterson 2015, Doolin et al. 2020).

Studies on drug filtration, reabsorption, and excretion in zebrafish embryos are hard to find. Generally, when drugs are administrated by continuous aqueous exposure through immersion, there should be a stable equilibrium between the absorption and excretion of the compound (Diekman and Hill 2013). Conversely, when drugs are injected into the zebrafish embryos, excretion studies can be performed using LC-MS pulsed exposure experiments. Once the compound is injected into the zebrafish, the internal compound concentration can be compared to the compound’s concentration in water daily to determine the approximate rate of excretion (Diekman and Hill 2013).

Zebrafish embryos have been extensively used for compound toxicology assessment. As a result of this, there is also a standard protocol for the zebrafish embryo acute toxicity test (ZFET) (European Commision 2013). Typical read-outs for acute toxicity are survival and developmental abnormalities upon drug exposure. Due to the transparency of the embryos, the abnormalities of exterior structures, like eyes and fins, as well as internal organs, like the heart and gut, can be assessed using microscopy. For example, the toxicity of anti-TB thiocarbamate compounds was assessed by investigating embryo mortality, hatching rate, heartbeat, and movement pattern (Aspatwar et al. 2017). In addition, histopathology of different tissues was performed in order to select the most promising derivative. Recently, beta-lactam antibiotics were investigated for their toxicity in a zebrafish embryo model by examining their malformation and lethality, which was followed by establishing a structure–toxicity relationship model for prediction of the acute toxicity (Han et al. 2021). Besides the examination of physiological features, also the behavior of embryos can be investigated. Different behavior models for assessing the effect of compounds on zebrafish embryos exist and are reviewed elsewhere (Rosa et al. 2022). Furthermore, detailed organ-specific toxicology studies can be performed using transgenic zebrafish lines, where embryos express fluorescent proteins using tissue- or cell-type specific promoters, allowing a quick determination of organ size or, e.g. the number of hepatocytes affected by toxicity in the liver (Cornet et al. 2017). Such fluorescence-based assays are also established to examine cardiotoxicity, neurotoxicity, and developmental toxicity as reviewed by Hill et al. (2005), McGrath and Li (2008), Scholz et al. (2008), and Eimon and Rubinstein (2009). Notably, the results from toxicology studies in zebrafish larvae are mostly in line with the ones performed in mammals (Ali et al. 2011, Ducharme et al. 2015, Vorhees et al. 2021).

## Evaluation of antibacterial compounds in zebrafish embryo infection models

The first studies using the zebrafish embryo model tested various clinically established antibiotics. These studies confirmed the applicability of this model for the *in vivo* evaluation of antimicrobials (Table S1, Supporting Information) (Adams et al. 2011, Bernut et al. 2014, Habjan et al. 2021, Knudsen Dal et al. 2022). Subsequently, several different pathogen-specific zebrafish infection models have been established for the evaluation of novel compounds (Table 2). The potency of different compounds can be studied by dose–response studies. Furthermore, this model can also be used to compare bacterial variants with altered drug susceptibility (Adams et al. 2011). In most publications, a zebrafish infection model was used to confirm antibacterial activity previously observed *in vitro* (Bernut et al. 2014, 2019, Johansen et al. 2021, Kim et al. 2021, Sullivan et al. 2021), although it would perhaps be more useful and exciting to test direct activity in zebrafish embryos. This would not only speed up the process but will also reveal compounds that would not have been identified in *in vitro* screenings, either because they are activated by the host metabolism, or because the metabolism of the pathogen inside the host differs from that in standard culture medium.

**Table 2.** An overview of novel antimicrobial and host-directed compounds and the synergistic combinations active in the zebrafish-embryo infection model. The table contains information about the compound’s name and mode of action and the bacteria sensitive to the compound. Moreover, the *in vitro* effective dose against the bacterial pathogen is specified next to the *in vivo* effective dose of the test compound in the zebrafish embryo infection model of the same pathogen (ZF) and the compound and pathogen administration route used in these zebrafish infection studies. Compounds designated with ‘*’ indicate those previously evaluated for antibacterial activity *in vitro* and subsequently confirmed using a zebrafish infection model. (Mmar = *M. marinum*; Mtb = *M. tuberculosis*).

Antimicrobial compound	Mode of action/PROPOSED Target	Sensitive bacteria	*In vitro* effective dose (MIC90)	*In vivo* (ZF) effective dose	Pathogen administration route (ZF)	Drug administration route (ZF)	Reference
Kalafungin*	Inhibition of β-lactamase and cell envelope disruption	*S. aureus* strain 6850	26.6 µM	26.6 µM	Caudal vein microinjection	Immersion	Mary et al. (2021)
Rhenium complexes*	Not specified	*S. aureus* MRSA43300	6.2 µM	25 µM	Caudal vein microinjection	Immersion	Sovari et al. (2020)
C23*	Not specified	methicillin resistant *S. aureus* (MRSA) (strain not specified)	2.5 µg/ml	10 µg/ml	Immersion	Immersion	Kannan et al. (2014)
Sorangicin A*	Inhibition of bacterial RNA polymerase	*S. aureus* Newman	78 nM	45 ng	Yolk microinjection	Yolk or caudal vein microinjection	Fries et al. (2023)
Epetraborole (EPT)*	Inhibition of Leucyl-tRNA synthetase	*M. abcessus* ATCC 19977	0.27 µM	42 µM	Caudal vein microinjection	Immersion	Kim et al. (2021), Sullivan et al. (2021)
disulfiram*	Not specified	M. abscessus ATCC 19977 *M. abcessus* MAB_010708_1655 (amikacin-resistant)	54–107 µM 54 µM	1 ng	Caudal vein microinjection	Posterior cardinal vein microinjection	Winters et al. (2022)
dithiocarbamate Fc14–584B*	β-CA-specific inhibition	*M. marinum* ATCC 927	75 µM	300 µM	Caudal vein microinjection	Caudal vein microinjection	Aspatwar et al. (2017)
PM-C7*, PM-C11*, and PM-C12*	Not specified	*M. marinum* M	0.06 µM (C7) 2 µM (C11) 0.9 µM (C12)	10 mg/kg (C7) 20 mg/kg (C11) 10 mg/kg (C12)	Posterior cardinal vein microinjection	Posterior cardinal vein microinjection	Knudsen Dal et al. (2022)
C2* and C4*	Not specified	EthA/KatG-overexpressing *M. marinum* M	0.44 µM (C2) 0.29 µM (C4)	2.5 µM (both C2 and C4)	Yolk microinjection	Immersion	Ho et al. (2021)
PBTZ169*	Inhibition of DprE1	*M. tuberculosi*s H37Rv *M. marinum* M	0.7 nM (Mtb H37Rv) 0.7 nM (Mmar)	25 nM	Caudal vein microinjection	Immersion	Makarov et al. (2014)
CCA34*	Inhibition of mycolic acid biosynthesis	*M. marinum* M M. tuberculosis H37Rv	0.75 µM	15 µM	Yolk microinjection	Immersion	Stanley et al. (2013)
TBA161-C*	Inhibition of Aspartyl tRNA synthase	*M. tuberculosis* H37Rv *M. marinum* M	1.3 ± 0.7 µM (Mtb) 1.3 ± 0.1 µM (Mmar)	0.3 µM	Yolk microinjection	Immersion	Habjan et al. (2021)
TBA8*	Proposed target: MmpL3	*M. tuberculosis* H37Rv *M. marinum* M	15.8 ± 7.5 µM (Mtb) MIC80 ≤ 10 µM (Mmar)	10 µM	Yolk microinjection	Immersion	Habjan et al. (2021)
TBA29*	Proposed target: Cytochrome bc1	*M. tuberculosis* H37Rv *M. marinum* M	3.6 ± 0.9 µM (Mtb) MIC80 ≤ 10 µM (Mmar)	3 µM	Yolk microinjection	Immersion	Habjan et al. (2021)
TBA32*	Proposed target: Thymidylate synthase	*M. tuberculosis* H37Rv *M. marinum* M	2.0 ± 0.5 µM (Mtb) MIC80 ≤ 10 µM (Mmar)	3 µM	Yolk microinjection	Immersion	Habjan et al. (2021)
TBA37*	Proposed target: MmpL3	*M. tuberculosis* H37Rv *M. marinum* M	13.6 ± 4.3 µM (Mtb) MIC80 ≤ 10 µM (Mmar)	10 µM	Yolk microinjection	Immersion	Habjan et al. (2021)
TBA38*	Proposed target: Cytochrome bc1	*M. tuberculosis* H37Rv *M. marinum* M	9.9 ± 1.9 µM (Mtb) MIC80 ≤ 10 µM (Mmar)	1 µM	Yolk microinjection	Immersion	Habjan et al. (2021)
TBA52*	Proposed target: MmpL3	*M. tuberculosis* H37Rv *M. marinum* M	9.6 ± 5.3 µM (Mtb) MIC80 ≤ 10 µM (Mmar)	10 µM	Yolk microinjection	Immersion	Habjan et al. (2021)
TBA57*	Proposed target: DprE1	*M. tuberculosis* H37Rv *M. marinum* M	1.7 ± 3.1 µM (Mtb) MIC80 ≤ 10 µM (Mmar)	1 µM	Yolk microinjection	Immersion	Habjan et al. (2021)
TBA61*	Proposed: Inhibitor of folate biosynthesis	*M. tuberculosis* H37Rv *M. marinum* M	2.2 ± 0.2 µM (Mtb) MIC80 ≤ 10 µM (Mmar)	1 µM	Yolk microinjection	Immersion	Habjan et al. (2021)
TBA117*	Proposed target: Cytochrome bc1	*M. tuberculosis* H37Rv *M. marinum* M	4.0 ± 1.4 µM (Mtb) MIC80 ≤ 10 µM (Mmar)	0.3 µM	Yolk microinjection	Immersion	Habjan et al. (2021)
TBA120*	Proposed target: Cytochrome bc1	*M. tuberculosis* H37Rv *M. marinum* M	5.9 ± 2.6 µM (Mtb) MIC80 ≤ 10 µM (Mmar)	0.3 µM	Yolk microinjection	Immersion	Habjan et al. (2021)
TBA139*	Proposed target: DprE1	*M. tuberculosis* H37Rv *M. marinum* M	11.8 ± 12.4 µM (Mtb) MIC80 ≤ 10 µM (Mmar)	3 µM	Yolk microinjection	Immersion	Habjan et al. (2021)
TBA145*	Proposed target: MmpL3	*M. tuberculosis* H37Rv *M. marinum* M	19.0 ± 5.7 µM (Mtb) MIC80 ≤ 10 µM (Mmar)	10 µM	Yolk microinjection	Immersion	Habjan et al. (2021)
TBA172*	Proposed: Inhibitor of folate biosynthesis	*M. tuberculosis* H37Rv *M. marinum* M	2.4 ± 0.4 µM (Mtb) MIC80 ≤ 10 µM (Mmar)	3 µM	Yolk microinjection	Immersion	Habjan et al. (2021)
GSK10*	Not specified	*M. tuberculosis* H37Rv *M. marinum* M	0.06 µM (Mtb) 0.39 µM (Mmar)	10 µM	Yolk microinjection	Immersion	Ordas et al. (2015)
GSK14*	Not specified	*M. tuberculosis* H37Rv *M. marinum* M	0.47 µM (Mtb) 0.39 µM (Mmar)	10 µM	Yolk microinjection	Immersion	Ordas et al. (2015)
GSK37*	Not specified	*M. tuberculosis* H37Rv *M. marinum* M	0.22 µM (Mtb) 12.5 µM (Mmar)	10 µM	Yolk microinjection	Immersion	Ordas et al. (2015)
GSK43*	Not specified	*M. tuberculosis* H37Rv *M. marinum* M	1.28 µM (Mtb) 6.25 µM (Mmar)	10 µM	Yolk microinjection	Immersion	Ordas et al. (2015)
SN30488*	Inhibition of mycolic acid synthesis (pretonamid derivative)	*M. tuberculosis* H37Rv *M. marinum* M	16.4 µg/l (Mtb) >4.2 mg/l (Mmar)	10 µM	Caudal vein microinjection	Immersion	Dalton et al. (2017)
SN30527*	Inhibition of mycolic acid synthesis (pretonamid derivative)	*M. tuberculosis* H37Rv *M. marinum* M	230 µg/l (Mtb) 3.7 mg/l (Mmar)	10 µM	Caudal vein microinjection	Immersion	Dalton et al. (2017)
Synergistic antimicrobial combinations							
L8S1 + Rifampicin*	Speculated outer membrane perturbing activity of L8S1	Clinical isolate *A. baumannii* 1757	0.612 µM (L8S1) + 0.171 µM (Rifampicin)	3.125 µM (L8S1) + 0.5 µM (Rifampicin)	Caudal vein microinjection	Caudal vein microinjection	Schouten et al. (2022)
l8s1 + 17fα*	Speculated outer membrane perturbing activity of L8S1 and inhibition of the FtsQB divisome complex by 17fα	Clinical isolate *E. coli* 87	0.9 µM (L8S1) + 3.9 µM (17fα)	3 125 µM (L8S1) + 70 µM (17fα)	Caudal vein microinjection	Caudal vein microinjection	Paulussen et al. (2022)
floxuridine + azidothymidine*	Speculated DNA damage	*E. coli* blood isolates BEC1–BEC8	< 0.04 (floxuridine) + <1.0 (azidothymidine)	0.19 µM (floxuridine) + 22 nM (azidothymidine)	Pericardial cavity microinjection	Yolk microinjection	Wambaugh et al. (2017)
Rifaximin + clarithromycin*	Inhibition of RNA polymerase by rifaximin (rifamycin derivative)	*M. abscessus*	Not specified	100 µM (Clarithromycin + 75 µM (rifaximin)	Hindbrain ventricle microinjection	Immersion	Goh et al. (2023)
Rifampicin + thiolactomycin (TLM)	Fatty acid and mycolic acid biosynthesis inhibition by TLM	*M. marinum* M *M. marinum* 4E4	Not specified	400 µM (TLM) + 100 µM (Rifampicin)	Caudal vein microinjection	Immersion	Takaki et al. (2012)
Host-directed compounds							
GSK1379760A*	Kinase PI3K/VPS34 inhibitor	*S. typhimurium* SL1344	Not active	10 µM	Duct of Cuvier microinjection	Immersion	van den Biggelaar et al. (2024)
Imatinib and isoniazid*	ABL tyroxine kinase inhibition	*M. marinum* M ATCC BAA–535	Not specified	100 µM (dexamethasone) 100 µM (acetylsalicylic acid)	Caudal vein microinjection	Immersion	Takaki et al. (2012)
DEXAMETHASONE*	Inhibition of the host’s enzyme leukotriene A4 hydrolase	*M. marinum ATCC BAA–535*	Not specified	0.75 µM	Caudal vein microinjection	Immersion	Takaki et al. (2012)
Acetylsalicylic acid*	Inhibition of the host’s enzyme leukotriene A4 hydrolase	*M. marinum ATCC BAA–535*	Not specified	1 µM	Caudal vein microinjection	Immersion	Takaki et al. (2012)
TMP195 and TSA*	Inhibition of host histone deacetylases	*M. tuberculosis* H37Rv *M. marinum* M	Not specified	10 µM (TMP195) 30 nM (TSA)	Duct of Cuvier microinjection	Immersion	Moreira et al. (2020)
CH-223191*	Inhibition of ligand-activated transcription factor aryl hydrocarbon receptor	*M. marinum* M *M. marinum* E11	Not specified	10 µM	Caudal vein microinjection	Immersion	Puyskens et al. (2020)
Desipramine	Inactivation of acid sphingomyelinase	*M. marinum* M	Not specified	7.5 µM	Caudal vein microinjection	Immersion	Roca and Ramakrishnan (2013)
Compound B*	OXSR1 inhibitor	*M. marinum M*	Not specified	1.8 µM	Microinjection	Immersion	Hortle et al. (2022)
Clemastine*	Purinergic receptor P2RX7	*M. marinum*	Not active	5 µM	Microinjection	Immersion	Matty et al. (2019)

In the zebrafish model of TB, several new compounds have been identified using the zebrafish model and *M. marinum* as a model organism. For example, putative TB drugs, such as compound PBTZ169 (Makarov et al. 2014) and mycolic acid biosynthesis inhibitor CCA34 (Stanley et al. 2013), were both active in zebrafish embryos as well as in mice models of TB. In addition, Aspatwar et al. (2017) reported about the β-CA-specific inhibitor dithiocarbamate Fc14–584B, which showed efficacy against *M. marinum* in infected zebrafish. Furthermore, Dalton et al. (2017) reported testing of antimycobacterial compounds in zebrafish embryos naturally infected by *M. marinum* through immersion. Besides showing the activity of known antibiotics like delamanid, pretonamid, and rifampicin, they also showed the activity of two novel pretonamid analogues SN30527 and SN30488. Another study described several zebrafish-active prodrugs (Ho et al. 2021), which were identified by using the *M. marinum* strain overexpressing *katG* and *ethA*, two common prodrug-activating enzymes. Furthermore, the efficacy of several nitronaphthofuran derivatives was investigated in a zebrafish–*M. marinum* model, where compounds were injected into the zebrafish posterior cardinal vein, which is the vein that follows the upper side of the yolk extension and leads to the caudal vein (Thisse et al. 2001). In this study, the investigated compounds were formulated in biocompatible polymeric micelles in order to improve their solubility. The authors compared different derivatives of compounds and selected the most potent ones (Knudsen Dal et al. 2022). Therefore, this study also showed that the zebrafish model can be used as a platform to study structure–activity relationships *in vivo*.

In the zebrafish embryos infected with *Mycobacterium abscessus*, two clinically established drugs, clarithromycin and imipenem, showed antimicrobial activity, thus validating the model for future drug screening purposes (Bernut et al. 2014). Furthermore, an *in vitro* drug screen identified a novel compound epetraborole, which was subsequently shown to be active in a zebrafish–*M. abscessus* model (Kim et al. 2021, Sullivan et al. 2021). Infections with *M. abscessus* are prevalent in patients with cystic fibrosis (CF), a genetic disease caused by a defective CF transmembrane conductance regulator (CFTR). To deplete the CFTR levels in zebrafish embryos, a morpholino-modified oligonucleotide (MO) was injected into the embryos to decrease expression and these CFTR-deficient zebrafish embryos were shown to mimic CF immunopathogenesis (Bernut et al. 2019). This zebrafish embryo model of CF was further used to assess the efficacy of bacteriophage treatment against *M. abscessus* infections (Johansen et al. 2021). Moreover, a recent study investigated the activity of the FDA-approved nonantibiotic drug disulfiram, which showed activity against drug-susceptible and amikacin-resistant *M. abscessus* infection in the zebrafish embryo model (Winters et al. 2022).

Recent papers used *S. aureus*-infected zebrafish to evaluate natural compounds for their antimicrobial activity. The activity of kalafungin, produced by *Streptomyces tanashiensis*, and the novel compounds C23 and ICN3 showed potent activity in *S. aureus*-infected zebrafish (Kannan et al. 2014, Mary et al. 2021). Likewise, various synthetic organometallic rhenium (Re) complexes were active in methicillin-resistant *S. aureus* (MRSA)-infected zebrafish (Sovari et al. 2020). Several other studies investigated compounds directed against *S. aureus* (Xiong et al. 2012, Stevens et al. 2015, Zhang et al. 2018, Dimer et al. 2020, Fenaroli et al. 2020) but have already been reviewed elsewhere (Rasheed et al. 2021). Moreover, various antibiotics were evaluated for their effectiveness in treating *S. aureus*-infected zebrafish embryos (Fries et al. 2023). Infection was induced through yolk injection, and the antibiotics were administered through caudal vein injection, yolk injection, or immersion. While reference antibiotics (ciprofloxacin, tetracycline, cefazolin, and vancomycin) proved effective in at least one administration method, notable differences were observed among the various routes of administration. Subsequently, the researchers explored the potential of sorangicin A (SorA), a natural compound with established *in vitro* activity. Microinjection of SorA into the yolk sac *of S. aureus-*infected embryos exhibited a significant increase in the survival rate and a reduction in bacterial burden, whereas the immersion method was ineffective.

Nogaret et al. (2021) exploited zebrafish embryos to develop an infection model of *P. aeruginosa*. The infection was established by immersing tail-injured embryos in a medium containing the *P. aeruginosa* wild-type PAO1 strain. They confirmed that the model could be used for compound evaluation by immersing infected embryos in a solution containing ciprofloxacin 2 h after infection. Next, they showed the *in vivo* activity of quorum sensing inhibitory molecule *N*-(2-pyrimidyl)butanamide (C11), confirming the previously observed *in vitro* activity.

To study the interaction between pathogens and their effect on the activity of drugs, Hattab et al. (2022) coinfected zebrafish embryos via swim bladder microinjections with *Candida albicans* and *P. aeruginosa*, two common opportunistic pathogens coinfecting lungs of CF patients (Hattab et al. 2022). They investigated the activity of the antifungal compound fluconazole (FLC) during zebrafish swim bladder infections and saw that FLC is more effective in treating *C. albicans–P. aeruginosa* coinfection than fungal monoinfection, suggesting that *P. aeruginosa* enhances the activity of FLC.

## Drug-screening strategies in zebrafish embryo infection models

Advances in automated injection procedures (Wang et al. 2007, Carvalho et al. 2011, Veneman et al. 2014) allow for a higher number of injected zebrafish embryos in a short time, and this opened the possibility for large-scale compound testing. Up to date, there have been two publications of antibacterial compound screening using automated robotic injection to establish zebrafish infection models. In both studies, the zebrafish embryos were infected in the yolk with fluorescent *M. marinum* using microinjection, whereas treatment was performed by immersion of the embryos into water containing a compound. Ordas et al. (2015) investigated the activity of a small set of 15 compounds from the GSK library of preclinical anti-TB hit compounds. The compounds were preselected based on their *in vitro* activity against *M. tuberculosis* and *M. marinum*. Of the 15 tested compounds, only four significantly reduced bacterial burden in infected embryos. Additionally, our laboratory screened 240 compounds from the TB Alliance library for their *in vivo* activity (Habjan et al. 2021). These compounds were also preselected based on their *in vitro* activity against *M. tuberculosis* and *M. marinum*. Interestingly, of the 240 compounds that were active *in vitro*, only 14 compounds showed activity in our zebrafish–*M. marinum* model, highlighting the importance of using *in vivo* models at the early stages of the drug-discovery pipeline. In this study, we further identified the target of one of the hits TBA161 to be Aspartyl tRNA synthase of mycobacteria. Furthermore, several derivatives of the hit compound TBA161 were tested and the zebrafish model was used to select the most promising variant, in a follow-up experiment investigating structure–activity relationships.

## Assessment of combination therapy in zebrafish embryo infection models

Several groups have used the zebrafish embryo infection model to investigate drug combinations *in vivo*. Our laboratory used zebrafish embryos infected with a clinical isolate of *A. baumannii* to investigate the antimicrobial activity of peptides and their interactions with known antibiotics (Schouten et al. 2022). Zebrafish embryos, 1-day-old, were infected with *A. baumannii* through microinjection of the caudal vein, followed by caudal vein microinjection of peptides or combinations of peptides and known antibiotics at 1 hpi. One of these peptides, stapled peptide L8S1, displayed synergistic activity with rifampicin, whereas its combination with erythromycin or vancomycin showed additive effects. This peptide was furthermore used to improve the efficacy of the novel antimicrobial compound 17fα and the combination was shown to act against *E. coli* infection in zebrafish embryos (Paulussen et al. 2022).

Drug combinations were also investigated in the *M. marinum*–zebrafish model, displaying the synergistic effect between rifampicin and isoniazid, similar to what is observed in the clinic (Takaki et al. 2012). In addition, Takaki et al. (2012) were able to show the synergy between rifampicin and thiolactomycin (TLM), which is a fatty acid biosynthesis inhibitor (Takaki et al. 2012). Moreover, in *M. abcessus-*infected zebrafish embryos the rifaximin was shown to potentiate the activity of clarihtomycin, which is currently the only highly effective oral antibiotic for the treatment of *M. abcessus* infections (Goh et al. 2023). A zebrafish–*E. coli* infection model was used to compare a standard treatment of trimethoprim and sulfamethizole to a newly proposed combination of floxuridine and azidothymidine (Wambaugh et al. 2017). Embryos were injected with a drug-sensitive *E. coli* strain, followed by treatment with drug combinations through injection, and both treatments performed similarly. However, when injected with trimethoprim-resistant *E. coli*, the new floxuridine–azidothymidine treatment showed 10 000-fold improved efficacy compared to the standard treatment.

## Exploration of host-directed approaches in zebrafish embryo infection models

Host-directed antimicrobial therapy is attracting attention in the drug development field, partly because it has been suggested to be less sensitive to bacterial resistance development (Kaufmann et al. 2018). However, compared to standard antibiotic treatments, host-directed therapy has a higher risk for adverse side effects (Tobin 2015). Moreover, the drug-discovery process can be challenging due to the limitations of current host models, such as cell lines. Zebrafish embryos present an interesting alternative by allowing for a whole-animal-based screening, bringing substantial advantages compared to the single-cell type tested in tissue culture. As mentioned previously, genetic manipulation of zebrafish to create transgenic lines is relatively easy, and several cell-type specific markers can be used to study the involvement of certain cell types or the immune defense responses (MacRae and Peterson 2015). Thus, using zebrafish embryos as a host model for host-directed therapies is as easy as testing in cell lines, while allowing modeling within the complexity of an entire system.

There are several reports of host-directed strategies, the majority using the zebrafish embryo model to prevent mycobacterial infections. Using a zebrafish–*M. marinum* infection model, Tobin et al. (2010) performed a forward genetic screen to identify genes involved in mycobacterial infection susceptibility. The authors first mutagenized a large population of zebrafish embryos using the chemical mutagen ethylnitrosourea (ENU). The mutagenized embryos were then raised to adulthood and bred to create a new library of zebrafish that carried random mutations in their genome. Their embryos were infected with *M. marinum* and further analyzed those that exhibited an increased resistance or susceptibility to infection. They subsequently genetically mapped the specific host genes that were responsible for the changes in infection susceptibility and found that the enzyme leukotriene A4 hydrolase (LTA4H) is critical in controlling mycobacterial infection (Tobin et al. 2010). Overexpression of the *lta4H* gene manifests in a hyperinflammatory state, resulting in increased mycobacterial growth. In a follow-up study using the hyperinflammatory zebrafish as hosts, dexamethasone and acetylsalicylic acid reduced the bacterial burden in the hyperinflammatory state (Tobin et al. 2012). This is an example of how studying and understanding the critical host response can assist in repurposing established host-directed drugs to control an infection.

Moreira et al. (2020) investigated if epigenetic features of the host genome control intracellular survival of *M. tuberculosis* in infected primary human macrophages, and they found the inhibition of host histone deacetylases (HDACs) as a potential host-directed therapy. They then showed that the pretreatment of zebrafish embryos with two different HDAC inhibitors (TMP195 and TSA) reduced *M. marinum* infection by more than 30% as compared to the nontreated control. Similarly, the ligand-activated transcription factor aryl hydrocarbon receptor (AhR) was investigated as a potential host target (Puyskens et al. 2020). AhR binds several antitubercular drugs, including rifampicin and rifabutin, resulting in altered host defence and faster drug metabolism promoting infection. However, adding the chemical inhibitor CH-223191 of AhR increased the activity of rifabutin in *M. marinum-*infected zebrafish embryos.

The study by Hortel et al. (2022) employed zebrafish embryos infected with *M. marinum* and *in vitro* THP-1 macrophage–*M. tuberculosis* systems to investigate the role of the WNK-OSXR1 signaling pathway in infection-induced inflammasome activation. The research demonstrated that pathogenic mycobacteria, particularly *M. marinum*, elevate macrophage K+ concentration and induce the expression of OXSR1. This induced OXSR1 was found to potentially suppress protective NLRP3 inflammasome responses and downstream IL-1β/TNF-α production. In the zebrafish infection model, it was observed that the virulent *M. marinum* induced the upregulation of both OXSR1 and SPAK, emphasizing the bacteria-driven modulation of host pathways for persistent infection. The study also demonstrated that small-molecule inhibition of OXSR1 activity mimicked the impact of OXSR1 knockdown on mycobacterial survival and could be a potential host-directed therapy against mycobacteria.

A recent study investigated ATP-competitive kinase inhibitors with known targets for their potential to be employed as host-directed therapies (van den Biggelaar et al. 2024). These investigations used intracellular infection models of *S. typhimurium* and *M. tuberculosis*. Initially, a screening process involved 825 compounds tested in infected human cell lines and primary macrophages. The selected hit compounds were investigated for *in vivo* toxicity and activity in the zebrafish embryo infection model. Two structurally related 2-anilino-4-pyrrolidinopyrimidines compounds showed activity in the *S. typhimurium*–zebrafish model.

These findings indicate the potential of utilizing this chemical scaffold as a form of host-directed therapy in the context of *Salmonella* infections. Conversely, no hit compounds were identified in the *M. marinum*–zebrafish infection model. The authors speculated that this discrepancy might arise from using *M. marinum* in the zebrafish model, whereas their initial screening was conducted in *M. tuberculosis* infection models.

While the mentioned studies used the zebrafish model to validate *ex vivo* findings, Matty et al. (2019) used the *M. marinum*-infected zebrafish to perform an unbiased host-directed screen of 1 200 FDA-approved compounds from the Prestwick Library. The 23 identified hit compounds were subsequently counter-screened for antibacterial activity in *in vitro* culture, leaving a selection of nine compounds with host-directed effects. Notably, one of the identified hits was desipramine, which had been previously proposed to be a potential host-directed compound (Roca and Ramakrishnan 2013), thus validating the described screening method. Another hit compound, clemastine, had earlier been reported to potentiate human P2 × 7 receptor (P2RX7) activity during cell tissue experiments (Norenberg et al. 2011). Since P2RX7 is known to act as a calcium channel, the effect of clemastine on the calcium dynamic within macrophages in zebrafish was investigated. They generated several transgenic zebrafish lines using a calcium reporter driven by a macrophage-specific promoter (Chen et al. 2013, Walton et al. 2015) and introduced it in a wild type and a *p2rx7* background. The embryos of the resulting zebrafish lines were exposed to clemastine. The wild type P2RX7 line showed a significant increase in the frequency of calcium flashes when compared to the nontreated group, whereas this effect was not seen in *p2rx7* mutants. Moreover, treatment with clemastine reduced the *M. marinum* burden in wild type zebrafish embryos but not in *p2rx7* mutants, indicating that clemastine functions as a host-directed compound that acts on the calcium channel P2RX7. This study demonstrates that zebrafish embryos can be used to screen and identify host-directed compounds and also to elucidate their mechanism of action. One caveat of studying host-directed therapies in zebrafish is that, due to the genetic distance between zebrafish and humans, the translational value of host-directed compounds is expected to be lower than antimicrobial compounds.

## Translational considerations in zebrafish embryo research

The ultimate question is how relevant the zebrafish embryo model is for predicting the clinical potential of antimicrobial compounds in humans. Can the zebrafish infection model compete with established murine models?

## Differences and similarities between zebrafish and mammalian models

An advantage of zebrafish as a model for the human infectious disease over invertebrate animals, such as *Caenorhabditis elegans* and *Galleria mellonella* (waxmoth) larvae, is its immune system. Overall, the zebrafish immune system is remarkably similar to that of humans, including both innate and adaptive immunity (Van der Sar et al. 2004, Meeker and Trede 2008). Additionally, 71% of human protein-encoding genes and 82% of disease-causing human genes have clear orthologues in the zebrafish genomes (Howe et al. 2013). Of course, the sequence similarity of the different immune receptors and mediators between humans and zebrafish is lower as compared to mice, but mice are not in all aspects better models. For example, it has been shown that there are significant differences in the inflammatory response between mice and humans (Mestas and Hughes 2004, Godec et al. 2016). Additionally, both zebrafish and mice have an organ system very similar to humans, including a liver, heart, pancreas, and intestines, but it can be difficult to model systemic infections in mice because of blood pressure differences, caused mainly by differences in resting heart rate between humans and mice (Hopper et al. 2021). In contrast, the cardiovascular physiology of zebrafish is similar to that of humans, allowing for systemic infections to be modelled with greater reliability and detail when compared to mice (MacRae and Peterson 2015).

A disadvantage of zebrafish is that they do not possess a bladder or lungs (MacRae and Peterson 2015, Barber et al. 2016). The absence of these organs may be problematic in investigating infectious diseases that physiologically occur in the lungs or the urinary tract, such as *M. tuberculosis* or uropathogenic *E. coli* (UPEC). Despite the lack of a urinary tract, UPEC still causes disease in zebrafish embryos when administered via injection (Barber et al. 2016). Moreover, TB progression can be investigated using *M. marinum*, the fish-born equivalent of *M. tuberculosis*, as a model pathogen (Bouz and Al Hasawi 2018). The infection of zebrafish with *M. marinum* manifests both systemically and in the formation of granulomatous lesions (Davis et al. 2002, Stoop et al. 2011), which is a hallmark of human infections with *M. tuberculosis*. Granulomas are aggregates of infected and noninfected immune cells, like macrophages, T-cells, B-cells, dendritic cells, and neutrophils as previously reviewed by (Philips and Ernst 2011, Ramakrishnan 2012, Russell 2013). The inner environment of granulomas can develop into a necrotic and hypoxic environment with high lipid content, termed caseum (Philips and Ernst 2011, Ramakrishnan 2012, Russell 2013). While *M. marinum* infections of adult zebrafish result in the formation of caseating granulomas (Swaim et al. 2006); infection of zebrafish embryos results in early stage granulomas, as was shown by the upregulation of special granuloma-activated gene markers of *M. marinum* (Davis et al. 2002). Bacteria within these early granulomatous lesions respond variably to drug treatment (Adams et al. 2011). This is also true for the antibiotics administered in the clinic, as some have better caseum penetration than others, and this is an important aspect of the efficacy of a drug in humans (Prideaux et al. 2015). Importantly, widely used mice strains C57BL6 and BALB/c do not mimic the formation of caseating granulomas (Kramnik and Beamer 2016).

A major shortcoming of zebrafish as a model for infectious diseases is their regenerative ability. While mammals have limited regenerative capacity, other vertebrates, including zebrafish, can regenerate a variety of body parts, including the heart, pancreas, liver, kidney, and musculoskeletal tissue as reviewed by (Gemberling et al. 2013, Daponte et al. 2021, Kaliya-Perumal and Ingham 2022; Ross Stewart et al. 2022). An inflammatory response hinders regeneration in mammals, while in zebrafish, it facilitates it (Iribarne 2021). This capacity allows zebrafish to survive beyond 24 hpf, even if they have organ abnormalities such as a malformed heart or tissue damage caused by *M. marinum* (Hill et al. 2005). Although interesting when investigating tissue regeneration, it can hinder the translational value of the model when characterizing antimicrobial compounds. Especially toxicological assessments can show aberrant results when zebrafish regenerate affected tissues. Finally, one obvious limitation of using zebrafish is the incubation temperature of 28°C, which often blunts the full expression of virulence genes by human pathogens that evolved at higher temperatures. Using closely related zebrafish pathogens is an effective method to circumvent this problem.

## Translational potential of antimicrobial testing in zebrafish embryos

Numerous drugs have been evaluated with the zebrafish embryo model and are now in clinical trials (Cully 2019, Patton et al. 2021). Additionally, many established drugs have been shown to be active in zebrafish retrospectively (Table S1, Supporting Information), accentuating the translational value of the zebrafish model in drug screening (Bouz and Al Hasawi 2018). Based on the reported studies, the *in vitro* activity of a tested compound seems to translate well to the *in vivo* zebrafish studies. However, it should be noted that experiments with negative outcomes are rarely published; thus, the positive outcomes might be over-represented. The differences between *in vitro* and *in vivo* activity can steer both ways; potential antimicrobials may show better *in vitro* activity than *in vivo* efficacy, but they can also appear more active *in vivo* compared to *in vitro* (Ordas et al. 2015, Habjan et al. 2021, Ho et al. 2021, Schouten et al. 2022). The former can likely be attributed to *in vivo* ADME properties (Diekman and Hill 2013), the latter, however, is not as easily explained. It has been suggested that compounds may interact synergistically with host molecules, such as host-specific antimicrobial peptides (AMPs) or defensive enzymes (Chen et al. 2019, Duong et al. 2021). For example, antimicrobials are known to exhibit synergistic activity with the human bacteriolytic enzyme lysozyme (Wittekind and Schuch 2016), and although such activity has not been investigated for zebrafish lysozyme, it likely occurs in the fish model as well. At 28 hpf, the most common time of infection by injection, lysozyme is expressed mainly within the caudal vein and thus circulates in the vascular system (Liu and Wen 2002). Another explanation for the discrepancy between *in vivo* and *in vitro* activity is the presence or absence of host enzymes that convert prodrugs (Ho et al. 2021), as was observed for the prodrug ethionamide. This prodrug is not active against *M. marinum in vitro*, even at 20 µM, but shows activity at 1 µM in *M. marinum*-infected zebrafish (Ho et al. 2021).

The two antimycobacterial drug screenings performed in a zebrafish infection model used preselected compounds with good *in vitro* activity and tested them in the zebrafish–*M. marinum* model (Ordas et al. 2015, Habjan et al. 2021). Both studies reported that less than 10% of the compounds active *in vitro* showed activity in the zebrafish infection model, which underscores the translational gap between *in vitro* and *in vivo* models. Ordas et al. (2015) additionally showed that for some of the compounds, the reason for inactivity was insufficient uptake by zebrafish. Of 15 compounds active *in vitro*, only four were active in the zebrafish model, and from those four only two displayed antimicrobial efficacies in a mice–*M. tuberculosis* infection model. On the other hand, from 11 compounds that were inactive in the zebrafish model, five did show antimicrobial activity in the mice model. This result highlights the considerable discrepancy between different models and the need to understand the reason behind it. Perhaps even more important is to determine how this data correlates with activity in humans and the zebrafish model could perhaps be further optimized by using specific transgenic zebrafish lines. For example, Poon et al. (2017) have developed a humanized zebrafish line, expressing the human CYP3A4 to alter the drug metabolism to be more comparable to humans in order to quickly conduct more relevant toxicity experiments.

While for novel compounds the difference in activity between models appears substantial, it is also clear that the translational potential of the zebrafish seems high when using established drugs approved for human use. An example is the treatment of *M. marinum-*infected zebrafish embryos with the anti-TB drug isoniazid. After assessing internal drug concentrations by nanoscale blood sampling and LC-MS/MS, the PK modeling showed that the isoniazid response against *M. marinum* in the zebrafish embryos correlated to the isoniazid response against *M. tuberculosis* in humans (Van Wijk et al. 2019). A similar study showed that uptake and clearance of paracetamol in zebrafish embryos correlated well with parameters found in higher vertebrates, including humans (Kantae et al. 2016, Van Wijk et al. 2019). The novel analytical methods used in these studies allowed for accurate measurements of the exposure–response relationship in zebrafish and hence improve the model’s predictive value for drug responses in humans.

Ordas et al. (2015) showed that the uptake of compounds by zebrafish embryos is a limiting factor for its activity. However, it remains difficult to determine or predict the uptake of these same drugs in humans. Our laboratory compared the activity of several clinically available antibiotics against *M. marinum, S. pneumoniae*, and *E. coli* in the zebrafish embryo infection model (Habjan et al. 2021). We noted that the antibiotics that are administered as intravenous or intramuscular injections in the clinic were only active when injected into the zebrafish bloodstream. Conversely, orally administered antibiotics were also active when infected embryos were incubated in a solution in which these antibiotics were dissolved. The successful treatment of infected embryos by immersion, therefore, seems to be selective for drugs with good oral bioavailability. However, since the mouth of zebrafish embryos does not open until 72 hpf, uptake is likely through diffusion through the skin.

Additionally, it should be noted that pharmaceutical companies are generally interested in the activity of a compound against infections with a high bacterial load. The zebrafish embryo infection models are unable to evaluate this activity since the number of infecting bacteria is generally low. Moreover, animal ethical regulations limit the experimental window. Likewise, we cannot discriminate between bacteriostatic and bactericidal compounds since we measure the inhibition of bacterial growth or death of the host. Different variations of zebrafish infection models are emerging in order to study different phenomena. For example, a study by Commandeur et al. (2020) reported a zebrafish embryo model of persistent mycobacteria within the available time-frame by using a specific resuscitation mutant of *M. marinum*. A previous study (Parikka et al. 2012) had reported that chronic infection of adult zebrafish with *M. marinum* resulted in the generation of a persistent *M. marinum* population dependent on functional resuscitation-promoting factors (Rpfs). Using this knowledge, Commandeur et al. (2020) infected zebrafish embryos with *M. marinum* Δ*rpfAB*, lacking two of those Rpfs, that were pregrown under nutrient-starving conditions. The mutants were able to establish an infection in the zebrafish embryos but retained a persister phenotype, like tolerating treatment with ethambutol and compromised growth. The zebrafish model with *M.marinum* Δ*rpfAB* mutant was proposed as a possible model for *in vivo* drug screening against mycobacterial persisters.

As mentioned, zebrafish can be genetically manipulated to study the impact of host genetic factors on susceptibility to infections. By introducing specific genetic variants associated with immune function, researchers can assess how variations in the host genome influence the response to pathogens. This information can be relevant for understanding individual susceptibility to infections and developing personalized prevention or treatment plans. Moreover, the ability to develop humanized zebrafish makes the zebrafish embryo model of great value in the field of personalized medicine as reviewed previously (Baxendale et al. 2017) and it can be hypothesized that the zebrafish embryo model may also be helpful in personalized antibiotic therapy decision making. In theory, the effectiveness of antibiotics in zebrafish infected with specific bacterial clinical isolates can be examined. However, utilizing the zebrafish embryo model as a tool for personalized antibiotic therapy decision-making currently appears challenging. This is due to the relatively short experimental period, around 6 days, and the labor-intensive nature of required techniques like manual microinjection. These constraints make it less competitive compared to clinical *in vitro* antibiotic activity testing.

Taken together, there are numerous reports validating the zebrafish-infection model as a valuable tool for compound screening and evaluation. However, it should also be noted that the experimental design varied greatly between them, which is linked to the type of pathogen and compound, i.e. studied. Nevertheless, even studies that use the same pathogen vary considerably with regards to injection timepoint, treatment timepoint, type of treatment, and duration of the treatment. Moreover, in some experiments, embryos receive treatment while they are still unhatched (Habjan et al. 2021), whereas in other cases, the embryos were manually dechorionated before the treatment started (Ordas et al. 2015). This makes it hard to compare the results of different studies and later translate them to other animal and clinical models.

## Optimization strategies to improve translation potential of zebrafish embryo models

There are several aspects that can be optimized to improve the translational value of the zebrafish model for antimicrobial drug screening. First, the relationship between *in vitro* and *in vivo* models should be established to compare drug activity profiles directly. This could be done by stating the compound’s active concentration in zebrafish based on the reported *in vitro* activity. Not only will this help in the translation between different models, but it will also serve as a guideline for future studies to decide on the starting test concentration. Future studies could use these data to evaluate how *in vitro* activity correlates with activity in zebrafish.

The introduction of a standardized protocol for performing drug efficacy testing using zebrafish embryos will allow for more accurate data comparisons from different research groups. For example, we propose a standardized incubation time between injection and treatment of zebrafish embryos, which we suggest to be defined as three times the pathogen’s replication time. For example, if zebrafish embryos are injected with *M. marinum*, the treatment will be performed at 21 hpi (replication time of 7 h), whereas when embryos are infected with *E. coli*, the treatment will be done at one hpi (replication time of 20 min). Since the experimental window of zebrafish embryo experiments is short, we suggest for studies that investigate potential host-directed compounds, a pretreatment of zebrafish embryos 1 day before infection to allow for a timely stimulation of the targeted host pathway. The optimal time for a potential host-directed drug to reach its target depends on the molecular characteristics of the compound, such as its hydrophobicity, the administration route, and the localization and properties of the host target. Nonetheless, based on the absorption and distribution studies published to date (Diekman and Hill 2013, Park et al. 2020), most compounds will likely activate a host response after 24 h of treatment.

**Figure 4.**
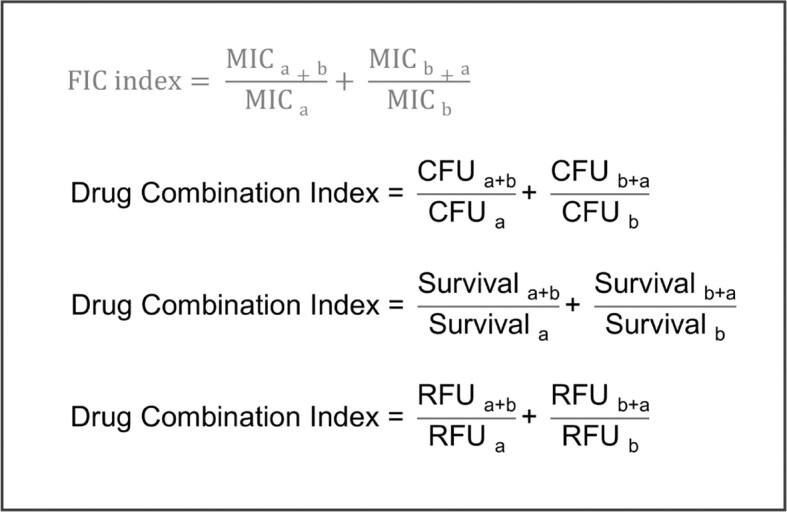
DCI formulas for quantification of drug-to-drug interactions in zebrafish embryo experiments. Shown are the DCI formulas based on the existing Fractional inhibitory concentration (FIC) index formula used for *in vitro* synergy testing, where a minimum inhibitory concentration (MIC) of drug *a* or *b* alone (MIC *a* or MIC *b*) is compared to the MIC of *a* and *b* combined (MIC *a* + *b*). DCI formula depends on the experimental read-out used in zebrafish embryo experiments, such as CFU, zebrafish embryo survival in percentage per treatment group, or mean relative fluorescence unit (RFU) per treatment group. The drug-to-drug interactions are interpreted from FICI and DCI values as: ≤ 0.5 = synergistic; 0.5–1.0 = additive; > 1.0–4.0 = indifferent; and > 4.0 = antagonistic.

In addition, clear guidelines are also needed to evaluate drug combinations in zebrafish embryos. *In vitro* interactions between drugs are typically quantified by a checkerboard assay followed by calculation of the fractional inhibitory concentration index (FICI) (Hsieh et al. 1993, Chou et al. 2019) (Fig. 4). The resulting FICI value defines synergy (FICI ≤ 0.5), an additive effect (FICI 0.5–1), indifference (1–4), or antagonism (FICI > 4) (Berenbaum 1980, Hsieh et al. 1993). Although this value can be used for *in vivo* studies, it is mostly used *in vitro* (Chou et al. 2019) and would require a considerable number of embryos to get a fully representative of MICs. In most zebrafish studies, statistical *P*-value analyses are used to determine synergy. For example, Takaki et al. (2012) tested synergy between rifampicin and isoniazid against *M. marinum* in zebrafish embryos and defined synergy as a bacterial burden, i.e. statistically lower than either one of the drugs alone, but it is difficult to determine whether the drug interaction is synergistic or additive. To improve the translational value of *in vivo* synergy studies, we suggest adapting the FICI calculation to quantify *in vivo* activity to discriminate synergistic, additive and antagonistic effects. We previously described *in vivo* synergy between membrane perturbing peptides and antibiotics by using zebrafish embryo survival counts as input for the FICI calculation (Schouten et al. 2022). Here, we propose to replace that index by the Drug Combination Index (DCI). The DCI can be calculated similarly to the FICI, by replacing the MIC values in FICI calculations for either survival percentages or mean fluorescence values or CFUs per treatment group, yielding the DCI that quantifies the activity of drug combinations in zebrafish embryos (Fig. 4). Using this calculation, we are indexing the fold-difference in the treatment outcome. The difference between a single drug and a combination of drugs needs to be higher than 4-fold to classify the combination as synergistic, while a 2-fold difference could be considered an additive combination. The use of the index would objectify the data of *in* vivo compound testing to a level observed with the *in vitro* data. Naturally, the DCI can also be used for synergy studies in other animal models.

## Conclusions

Zebrafish embryos are now accepted in the field as an attractive model to study infections with different pathogens as well as to characterize compounds and pharmaceuticals. By combining these methods, it is possible to use pathogen-infected zebrafish embryos to screen, identify and evaluate antimicrobial compounds. The protocols for establishing the zebrafish embryo infection model can be adapted in many ways to suit the purpose of a specific study and can be used for medium-throughput screening of antimicrobials, or to evaluate early stage compounds for their ADME properties and their toxicity. Furthermore, the mechanisms of action and drug combinations can be studied in detail. The model can be used to perform structure–activity relationship studies and select the most promising lead compound. It also allows for the identification and characterization of prodrugs and host-directed compounds. Thus far, most studies are done using zebrafish–*M. marinum* infection model, whereas there is limited literature on compound testing in zebrafish embryos infected with other Gram-positive and Gram-negative bacteria. Accordingly, there is a research gap that will hopefully be filled in the future by reports using a variety of pathogens. Furthermore, there are only a few reports on the screening of antimicrobials on a large scale. However, with the advances in technology like automated injection, treatment, imaging, and analyzing techniques, the throughput of drug testing in zebrafish will increase, allowing for screening of large libraries of compounds. Consequently, the zebrafish *in vivo* screening platform could be used early in the drug discovery pipeline. As such, it will not only serve as a bridge between *in vitro* assays and *in vivo* mammalian studies but also as a first-line screening strategy to identify *in vivo* active compounds. This will allow for rapid, economical, and efficient identification of active and nontoxic compounds and hopefully aid in the success rate of selected hits during later clinical studies. As with every model, also the zebrafish infection model has its advantages and limitations (Table 3). Some of the limitations will hopefully be solved in the future by the development of novel techniques and assays. Even though the field is rapidly evolving, extensive knowledge of the subject is needed to interpret the phenomena observed in the zebrafish model. Taken together, the zebrafish infection model can be used as a cost and time-effective model for antimicrobial drug screening and characterization. As evidence accumulates, the translational value of the model will increase. Nevertheless, the standardization of the protocols and further progress in understanding drug PKs in zebrafish will allow this *in vivo* model to reach its full potential for early stage antimicrobial evaluation.

**Table 3.** A summary of advantages, disadvantages, and biases of zebrafish embryo infection model in antimicrobial drug screening.

	Advantages	Disadvantages	Biases
**Genetics**	Most human genes have obvious orthologues in zebrafish.	Gene duplications in zebrafish make it hard to identify orthologue and can complicate the generation of knockout/in zebrafish lines. The percentage similarity of immune receptors and effectors is generally low.	
	Transgenic lines offer the option of live-imaging of host responses to compounds or pathogens.		
**Handling**	Small size; relatively easy maintenance and breeding.	Maintained at 28–30°C, whereas mammalian pathogens are adapted to 37°C and are attenuated. Therefore, sometimes related fish pathogens are used.	
	Limited ethical restrain up to 5 dpf.	Experiments can only be tested in the first days of infection with replicating pathogens, which makes it difficult to test for chronic infections.	
	High fidelity allows for rapid and large egg production.		
**Physiology**	Cardiovascular physiology is similar to that of humans (more so than for murine models).	Absence of lungs and bladder.	
		Regenerative ability can hinder toxicology testing.	Compounds will reach the target tissue, and the metabolism and excretion are not fast.
	The immune system is similar to that of humans.	Lack of monoclonal antibodies for zebrafish immune cells or effectors.	
**Screening**	Time and cost-efficient experiments.	Potentially laborious experiments.	
		Challenging to study specific ADME properties individually.	Compound uptake is sufficient to observe activity and adverse effects.
		No standardized protocols, high variation in study design between different studies.	
**Read out**	Transparency allows for rapid examination of developmental abnormalities.	Challenging to perform tissue-specific analysis.	No standardized protocols, high variation in study design between different studies.
	Simultaneously evaluation of compounds activity and toxicity.	Relatively high variability compared to *in vitro* or *ex vivo* models.	Fish-born equivalent pathogens are similar to human pathogens.
	Host to study host factors and host-directed compounds.	Hard to establish a drug’s exposure–response relationship.	
	Host to study virulence factors and virulence inhibitors.	Challenging determination of internal drug concentration and drug distribution.	

## Supplementary Material

fuae011_Supplemental_File
