# Supplementary material for: Diving into drug-screening: zebrafish embryos as an in vivo platform for antimicrobial drug discovery and assessment
Source: FEMS Microbiol Rev. 2024 Apr 29;48(3):fuae011. doi: 10.1093/femsre/fuae011 (PMC11078164; doi:10.1093/femsre/fuae011)
Supplement: fuae011_Supplemental_File [file fuae011_supplemental_file.docx]

**Table S1**: Examples of clinically used antibiotics and their activity in the zebrafish-embryo infection model. (ZF = zebrafish infection model)

| aNTIBIOTICS TESTED IN ZEBRAFISH EMBRYOS RETROSPECTIVELY | MODE OF ACTION / PROPOSED TARGET | SENSITIVE BACTERIA | *In vivo* (ZF)  effective dose | DRUG ADMINISTRATION ROUTE (ZF) | REFERENCES |
| --- | --- | --- | --- | --- | --- |
| RIFAMPICIN | Inhibition of RNA polymerase | *M. tuberculosis* H37Rv  *M. marinum* M | 10 µM | Immersion | (Habjan, Ho et al. 2021) |
| SUTEZOLID | Inhibition of ribosomes | *M. tuberculosis* H37Rv  *M. marinum* M | 10 µM | Immersion | (Habjan, Ho et al. 2021) |
| BEDAQUILINE | Inhibition of ATP synthase | *M. tuberculosis* H37Rv  *M. marinum* M | 10 µM | Immersion | (Habjan, Ho et al. 2021) |
| ETHIONAMIDE | Enoyl-acyl carrier protein reductase InhA (mycolic acid synthesis) | *M. tuberculosis* H37Rv  *M. marinum* M | 5 µM | Immersion | (Habjan, Ho et al. 2021) |
| DELAMANID | Inhibition of mycolic acid synthesis | *M. tuberculosis* H37Rv  *M. marinum* M | 10 µM | Immersion | (Habjan, Ho et al. 2021) |
| PRETONAMID | Inhibition of mycolic acid synthesis | *M. tuberculosis* H37Rv  *M. marinum* M | 10 µM | Immersion | (Habjan, Ho et al. 2021) |
| SQ109 | Multi-mode action (inhibition of cell wall synthesis and energy production) | *M. tuberculosis* H37Rv  *M. marinum* M | 10 µM | Immersion | (Habjan, Ho et al. 2021) |
| LINEZOLID | Inhibition of ribosomes | *M. tuberculosis* H37Rv  *M. marinum* M | 40 µM | Immersion | (Habjan, Ho et al. 2021) |
| LEVOFLOXACIN | Inhibition of DNA gyrase and topoisomerase IV | *M. tuberculosis* H37Rv  *M. marinum* M | 130 µM | Immersion | (Habjan, Ho et al. 2021) |
| ETHAMBUTOL | Arabinosyltransferase EmbB inhibition (cell wall synthesis) | *M. tuberculosis* H37Rv  *M. marinum* M | 1442 µM | Immersion | (Adams, Takaki et al. 2011) (Habjan, Ho et al. 2021) |
| MOXIFLOXACIN | Inhibition of DNA gyrase and topoisomerase IV | *M. tuberculosis* H37Rv  *M. marinum* M | 62.3 µM | Immersion | (Adams, Takaki et al. 2011) |
| ISONIAZID | Inhibition of mycolic acid synthesis (InhA) | *M. tuberculosis* H37Rv  *M. marinum* M | 290 µM | Immersion | (Adams, Takaki et al. 2011) |
| LEVOFLOXACIN | Inhibition of DNA gyrase and topoisomerase IV | *E. coli* GSK1161434 | 9 µM | Immersion | (Habjan, Ho et al. 2021) |
| CEFTAZIDIME | Peptidoglycan synthesis inhibition | *E. coli* GSK1161434 | 0.2 µM | Caudal vein microinjection | (Habjan, Ho et al. 2021) |
| LEVOFLOXACIN | Inhibition of DNA gyrase and topoisomerase IV | *S. pneumoniae* D39V | 550 µM | Immersion | (Habjan, Ho et al. 2021) |
| PENICILLIN | Inhibition of penicillin-binding protein (PBP) | *S. pneumoniae* D39V | 17 µM | Immersion | (Habjan, Ho et al. 2021) |
| CEFTRIAXONE | Peptidoglycan synthesis inhibition | *S. pneumoniae* D39V | 0.05 µM | Caudal vein microinjection | (Habjan, Ho et al. 2021) |
| MEROPENEM | Inhibition of penicillin-binding protein (PBP) | *S. pneumoniae* D39V | 0.3 µM | Caudal vein microinjection | (Habjan, Ho et al. 2021) |
| CEFTAZIDIME | Peptidoglycan synthesis inhibition | *S. pneumoniae* D39V | 10 µM | Caudal vein microinjection | (Habjan, Ho et al. 2021) |
| Linezolid | Inhibition of ribosomes | *S. aureus*Newman | Yolk and caudal vein 37.5 ng  Immersion 100 µg/ml | Caudal vein or yolk microinjection or immersion | (Fries, Kany et al. 2023) |
| ciprofloxacin | Inhibition of DNA gyrase and topoisomerase IV | *S. aureus*Newman | 45 ng | Caudal vein or yolk microinjection | (Fries, Kany et al. 2023) |
| tetracycline | Inhibition of protein synthesis (30S ribosomal subunit) | *S. aureus*Newman | 30 ng | Caudal vein or yolk microinjection | (Fries, Kany et al. 2023) |
| cefazolin | Inhibition of penicillin-binding protein (PBP) | *S. aureus*Newman | 45 ng | Caudal vein or yolk microinjection | (Fries, Kany et al. 2023) |
| vancomycin | Inhibition of bacterial cell wall synthesis | *S. aureus*Newman | 30 ng | Yolk microinjection | (Fries, Kany et al. 2023) |
| AMIKACIN | Inhibition of protein synthesis (30S ribosomal subunit) | *M. abscessus* ATCC 19977 | 1.5 ng | Posterior cardinal vein microinjection | (Winters, Basnet et al. 2022) |
